# Huanglongmycin A-C, Cytotoxic Polyketides Biosynthesized by a Putative Type II Polyketide Synthase From *Streptomyces* sp. CB09001

**DOI:** 10.3389/fchem.2018.00254

**Published:** 2018-06-26

**Authors:** Lin Jiang, Hong Pu, Jingxi Xiang, Meng Su, Xiaohui Yan, Dong Yang, Xiangcheng Zhu, Ben Shen, Yanwen Duan, Yong Huang

**Affiliations:** ^1^Xiangya International Academy of Translational Medicine, Central South University, Changsha, China; ^2^Department of Chemistry, The Scripps Research Institute, Jupiter, FL, United States; ^3^Hunan Engineering Research Center of Combinatorial Biosynthesis and Natural Product Drug Discovery, Changsha, China; ^4^Department Molecular Medicine, The Scripps Research Institute, Jupiter, FL, United States; ^5^Natural Products Library Initiative, The Scripps Research Institute, Jupiter, FL, United States; ^6^National Engineering Research Center of Combinatorial Biosynthesis for Drug Discovery, Changsha, China

**Keywords:** nonaketide, type II polyketide synthase, karstic cave, huanglongmycin, cytotoxicity

## Abstract

Three natural products of nonaketide biosynthetic origin, probably biosynthesized from nine molecules of malonyl-CoA, have been isolated. Herein we described the isolation and structure elucidation of huanglongmycin (HLM) A-C and identification of the putative *hlm* biosynthetic gene cluster from *Streptomyces* sp. CB09001, isolated from a karstic cave in Xiangxi, China. Albeit previously isolated, HLM A was reported for the first time to exhibit moderate cytotoxicity against A549 lung cancer cell line (IC_50_ = 13.8 ± 1.5 μM) and weak antibacterial activity against gram-negative clinical isolates. A putative biosynthetic pathway for HLM A, featuring a nonaketide-specific type II polyketide synthase, was proposed. It would be consistent with the isolation of HLM B and C, which are two new natural products and likely shunt metabolites during HLM A biosynthesis.

## Introduction

**Figure 1:**
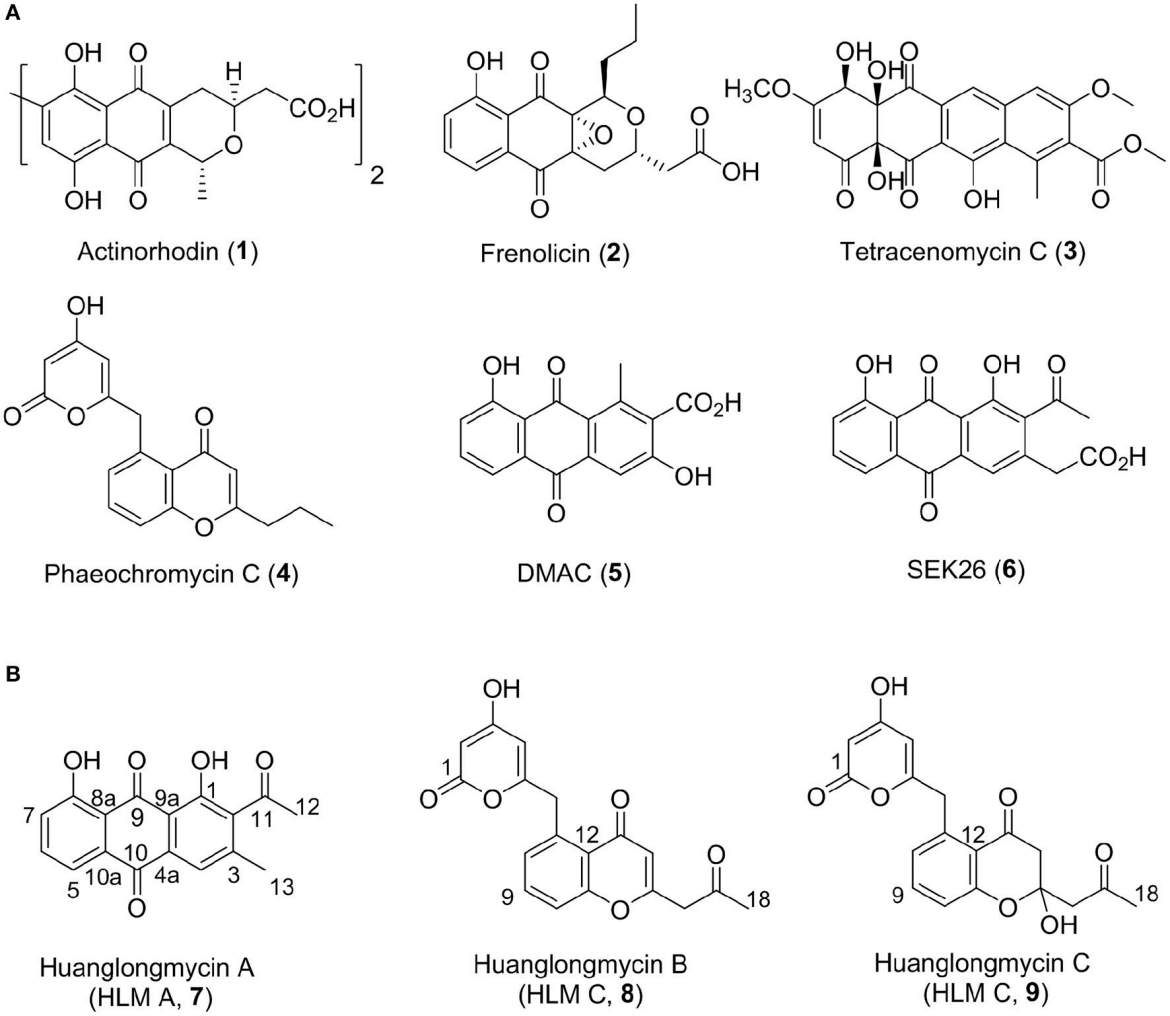
Structures of selected aromatic polyketides and their biosynthetic shunt products resulting from combinatorial biosynthesis (**1**–**6**) **(A)** and the three huanglongmycin A–C (**7–9**) isolated from *S*. sp. CB09001 **(B)**.

Aromatic polyketides of microbial origin are a large family of natural products with important biological activities, including anticancer agents doxorubicin and mithramycin, and antibiotics tetracyclines. Aromatic polyketides are typically biosynthesized by type II polyketide synthases (PKSs), minimally consisting of two ketosynthases KS_α_ and KS_β_ (also named “chain length factor”) and an acyl carrier protein (ACP), that are associated with additional ketoreductases and cylases/aromatases (Hopwood, 1997; Shen, 2000; Hertweck et al., 2007; Das and Khosla, 2009; Zhou et al., 2010; Zhang et al., 2017). The KS_α_, KS_β_, and ACP are often clustered together and called minimal PKSs. Extensive biosynthetic studies of these polyketides, such as actinorhodin (**1**), frenolicin (**2**), and tetracenomycin C (**3**), have helped establish certain “design rules” to make designer analogs, such as DMAC (**5**) and SEK26 (**6**), by a biotechnology platform now often termed “combinatorial biosynthesis” (Figure 1) (McDaniel et al., 1993, 1995; Kramer et al., 1997; Yu et al., 1998). However, only a few natural nonaketide-derived aromatic polyketides, including the biaryl compounds julichromes (**11**), setomimycin and spectomycins, have been proposed to be biosynthesized by the Claisen-like condensation of nine malonyl-CoA (Marti et al., 2000; Zhang et al., 2008; Präg et al., 2014). Interestingly, the nonaketide precursors could form homo- or heterodimers through oxidative phenol coupling (Präg et al., 2014).

We have been interested in collecting diverse microbial strains for natural product discovery and recently isolated several new natural products with antibacterial and anticancer activities (Luo et al., 2014; Ma et al., 2015, 2017; Yan et al., 2016; Pan et al., 2017). The western part of Hunan province (also named “Xiangxi”) in China is on the east of Yunnan-Kweichow plateau, which was geologically formed through epeirogeny uplifts in the late Permian period about 200-million years ago (Luo et al., 2014). Xiangxi is well-known for its characterized karstic topography and biodiversity (Chen et al., 2015). In this report, we describe the isolation, structure elucidation, biosynthesis, and biological activities of three aromatic polyketides from strain *S*. sp. CB09001, isolated from a karstic cave in Xiangxi (Figure 1).

## Materials and methods

### General experimental procedures

IR spectra were recorded on Nicolet iS50 FT-IR (Thermo Scientific). CD spectra were recorded on J-815 from JASCO. HRMS spectra were recorded on a LTQ-ORBITRAP-ETD instrument. NMR spectra were acquired using a Brucker 400 or 500 MHz spectrometer. Chemical shifts were reported in ppm relative to CDCl_3_ (δ = 7.26 ppm) or DMSO-*d*_6_ (δ = 2.50 ppm) for ^1^H NMR and CDCl_3_ (δ = 77.23 ppm) or DMSO-*d*_6_ (δ = 39.60 ppm) for ^13^C NMR spectroscopy. Column chromatography (CC) was carried out on silica gel (100–200 mesh and 300–400 mesh, Yantai Jiangyou Silica Gel Development Co., Ltd., Yantai, China) and polyamide (100–200 mesh, Sinopharm Chemical Reagent Co., Ltd., Shanghai, China). Semipreparative reversed phase-high-performance liquid chromatography (RP-HPLC) was performed using a Waters 1525 Binary HPLC Pump equipped with a Waters 2489 UV/Visible Detector and using a Welch Ultimate AQ-C18 column (250 × 10 mm, 5 μm).

### Bacterial strains

The *S*. sp. CB09001 strain was grown on G1 agar (2% soluble starch, 0.1% KNO_3_, 0.05% K_2_HPO_4_, 0.05% MgSO_4_·7H_2_O, 0.05% NaCl, 0.001% FeSO_4_·7H_2_O, 1.5% agar) plates and incubated at 28°C to obtain spores. For the genomic DNA isolation, *S*. sp. CB09001 was cultured in 250-mL baffled flasks containing 50 mL TSB liquid medium with 0.5% glycine and cultivated at 28°C for 36 h. Genomic DNA was isolated using standard protocols (Kieser et al., 2000). The 16S rRNA of *S*. sp. CB09001 was PCR-amplified and sequenced. The genome of *S*. sp. CB09001 was sequenced using the combination of Illumina Miseq/Hiseq and Pacific RSII platforms (Shanghai Majorbio Co., Ltd., Shanghai, China).

### Fermentation

The strain *S*. sp. CB09001 was grown in 250 mL Erlenmeyer flasks containing 50 mL of G1 medium and were incubated at 30°C on rotary shakers (230 rpm) for 72 h. Then 3% macroporous resin DA201-H (Jiangsu Su Qing Water Treatment Engineering Group Co., Ltd., Jiangyin, China) or different concentration triclosan (0–20 μM) were added, and fermented for additional 4 days. *S*. sp. CB09001 was also fermented in G1 fermentation medium with different fermentation volume in 250-mL flat-bottom Erlenmeyer flasks from 25 to 150 mL per flask. In scale-up fermentation, each of the seed cultures in G1 medium (50 mL) was aseptically transferred to 2 L Erlenmeyer flasks containing 1 L of G1 medium. All of these flasks were then incubated at 30°C on rotary shakers (200 rpm) for 7 days.

### Extraction and isolation

After fermentation, the culture (6 L) was centrifuged (4,000 rpm, 10 min) to yield the supernatant. After its pH was adjusted to 0.9 using concentrated HCl, the supernatant was extracted with EtOAc (3 × 6 L), which was concentrated under reduced pressure and subjected to silica gel CC using petroleum ether (PE)/EtOAc and EtOAc/MeOH containing 1% formic acid. Fractions A2-A3 (PE/EtOAc 90/10-80/20) was further purified by silica gel CC eluting with PE/EtOAc (90/10) to give three fractions. The first fraction was applied to silica gel CC eluted with CH_2_Cl_2_/MeOH (95/5) to obtain compound **7** (5.5 mg), which was further purified by recrystallization. Fractions A12-A15 (EtOAc/MeOH 90/10-60/40) were combined and subjected to silica gel CC using gradient elution with CH_2_Cl_2_/MeOH. The fractions (CH_2_Cl_2_/MeOH 92/8-90/10) were combined and subjected to polyamide CC using gradient elution with CH_2_Cl_2_/MeOH containing 1% formic acid (100/0, 94/6, 92/8, 90/10, 88/12, 86/14, 84/16, 80/20, 70/30, 60/40, 50/50, 40/60, 20/80, 0/100). The fraction C6 (86/14) was further purified by semipreparative RP-C18 HPLC (Welch Ultimate AQ 5 μm, 250 × 10 mm) with a flow rate of 3 mL/min and a gradient elution of CH_3_CN/H_2_O (containing 0.2% formic acid) in 20 min (5 to 95% for 15 min, followed by 95% for 2 min, and 95 to 5% for 0.5 min, followed by 5% for 2.5 min), to afford compounds **8** (*t*_R_ = 12.0 min, 5.3 mg) and **9** (*t*_R_ = 11.8 min, 33.3 mg), respectively.

**Huanglongmycin A (7):** Yellowish-brown solid; UV (MeOH) λmax (Abs) 192.8 (0.268), 225.8 (0.388), 255.4 (0.300), 428.7 (0.144); ^1^H, ^13^C, and 2D NMR spectroscopic data, see Table 1; (−)-HRESIMS *m*/*z* 295.0619 [M - H]^−^ (calcd. for C_17_H_11_O_5_, 295.0612).
**Huanglongmycin B (8):** Light yellow needle crystal; UV (MeOH) λmax (Abs) 200.4 (0.473), 229.8 (0.297), 298.6 (0.200); IR(νmax) 3,110, 2,960, 1,730, 1,710, 1,640, 1,590, 1,570, 1,480, 1,400, 1,320, 1,290, 1,160 cm^−1^; ^1^H, ^13^C, and 2D NMR spectroscopic data, see Table 1; (+)-HRESIMS *m*/*z* 327.0869 [M + H]^+^ (calcd. for C_18_H_15_O_6_, 327.0863), *m*/*z* 349.0687 [M + Na]^+^, *m*/*z* 653.1659 [2M + H]^+^, *m*/*z* 675.1480 [2M + Na]^+^.
**Huanglongmycin C (9):** Light yellow solid; UV (MeOH) λmax (Abs) 199.2 (0.488), 257.0 (0.170), 287.9 (0.142); IR(νmax) 3,440, 2,950, 1,650, 1,440, 1,310, 1,030 cm^−1^; ^1^H, ^13^C, and 2D NMR spectroscopic data, see Table 1; (+)-HRESIMS m/z 367.0794 [M + Na]^+^ (calcd. for C_18_H_16_O_7_Na, 367.0788), *m*/*z* 327.0870 [M - H_2_O + H]^+^, *m*/*z* 689.1872 [2M + H]^+^.
**Cytotoxicity assay:** Compounds **7–9** were evaluated for their cytotoxicity against the human cancer cell lines A549, SKOV3, Hela, and Caco-2 using the standard MTT method (Tai et al., 2014).
**Antibacterial assay:** Compounds **7–9** were tested for their antibacterial activities against *Staphyloccocus aureus* ATCC 29213, methicillin-sensitive *Staphylococcus aureus*, methicillin-resistant *Staphylococcus aureus, Escherichia coli, Klebsiella pneumoniae*, and *Pseudomonas aeruginosa* using a previously described method (Wiegand et al., 2008).

**Table 1:** NMR Spectroscopic Data for huanglongmycin A (HLM A, **7**), huanglongmycin B (HLM B, **8**) and huanglongmycin C (HLM C, **9**).

**Huanglongmycin A**				**Huanglongmycin B**				**Huanglongmycin C**			
**Position**	**δ_C_, type**	**δ_H_, mult. (*J* in Hz)**	**HMBC**	**Position**	**δ_C_, type**	**δ_H_, mult. (*J* in Hz)**	**HMBC**	**Position**	**δ_C_, type**	**δ_H_, mult. (*J* in Hz)**	**HMBC**
1	159.5, C			1	163.8, C			1	164.8, C		
2	136.2, C			2	88.3, CH	4.76, d (1.6)	1, 3, 4	2	88.0, CH	5.01, s	3, 4, 5
3	145.6, C			3	170.5, C			3	173.6, C		
4	122.2, CH	7.60, s	4a, 9a, 10, 13	4	99.5, CH	5.25, s	2, 5	4	101.9, CH	5.46, s	1, 2, 3, 6
4a	133.1, C			5	165.9, C			5	165.2, C		
5	120.2, CH	7.76, d (7.4)	7, 8a	6	37.4, CH_2_	4.37, s	4, 5, 7, 8, 12	6	37.7, CH_2_	4.08, d (16.0); 4.17, d (16.0)	5, 7
6	137.4, CH	7.64, m	8, 10a	7	135.9, C			7	135.5, C		
7	124.9, CH	7.24, d (7.8)	5	8	128.9, CH	7.29, d (7.4)	6, 10, 12	8	125.2, CH	6.95, d (8.3)	6, 10, 12
8	162.6, C			9	133.7, CH	7.71, m	7, 10, 11	9	136.9, CH	7.50, m	7, 11
8a	115.8, C			10	118.2, CH	7.51, d (8.3)	8, 9, 11, 12	10	118.3, CH	6.91, d (7.4)	8, 11, 15
9	192.6, C			11	157.6, C			11	159.4, C		
9a	114.1, C			12	121.0, C			12	118.9, C		
10	181.4, C			13	178.4,C			13	193.0,C		
10a	133.5, C			14	113.3, CH	6.22, s	12, 13, 15, 16	14	47.9, CH_2_	3.04, d (15.0); 3.12, d (15.0)	13, 15
11	202.8, C			15	162.2, C			15	100.5, C		
12	31.9, CH_3_	2.55, s	2, 11	16	47.6, CH_2_	3.96, s	14, 15, 17	16	52.4, CH_2_	2.73, d (15.4); 3.20, d (15.4)	13, 17
13	20.3, CH_3_	2.33, s	2, 3, 4	17	202.7, C			17	205.6, C		
1-OH		12.28, s	1, 2, 9a	18	30.0, CH_3_	2.24, s	16, 17	18	32.2, CH_3_	2.20, s	16, 17
8-OH		11.90, s	7, 8, 8a	3-OH		8.30, s		3-OH		8.23, s	

### Accession numbers

The 16S rRNA sequence and genome sequence of *S*. sp. CB09001 have been submitted to GenBank with accession number MG890330 and CP026730, respectively.

## Results and discussion

### Isolation and structure elucidation of huanglongmycins

We collected several soil samples from Huanglong cave in Xiangxi, from which new actinomycetes strains were isolated. One of the strains, CB09001, was classified as a *Streptomyces* species based on its morphological characteristics and the 16S rRNA sequence (NCBI accession no. MG890330). Encouraged by its unique metabolite profiles, *S*. sp CB09001 was fermented under various fermentation conditions, such as the addition of elicitor triclosan, and the variation of the fermentation volume and the addition of macroporous resin (Figures S1–S3) (Bode et al., 2002; Yoon and Nodwell, 2014; Tanaka et al., 2017). For large scale fermentation, *S*. sp. CB9001 was fermented in six 2-L Erlenmeyer flasks, containing 1 L of G1 medium, and isolation of natural products from the fermentation culture afforded three compounds, we named huanglongmycin (HLM) A–C (**7–9**). Their structures were established on the basis of HR-ESI-MS and extensive 1D and 2D NMR analysis (Figure 2, Figures S4–S25, Table 1).

**Figure 2:**
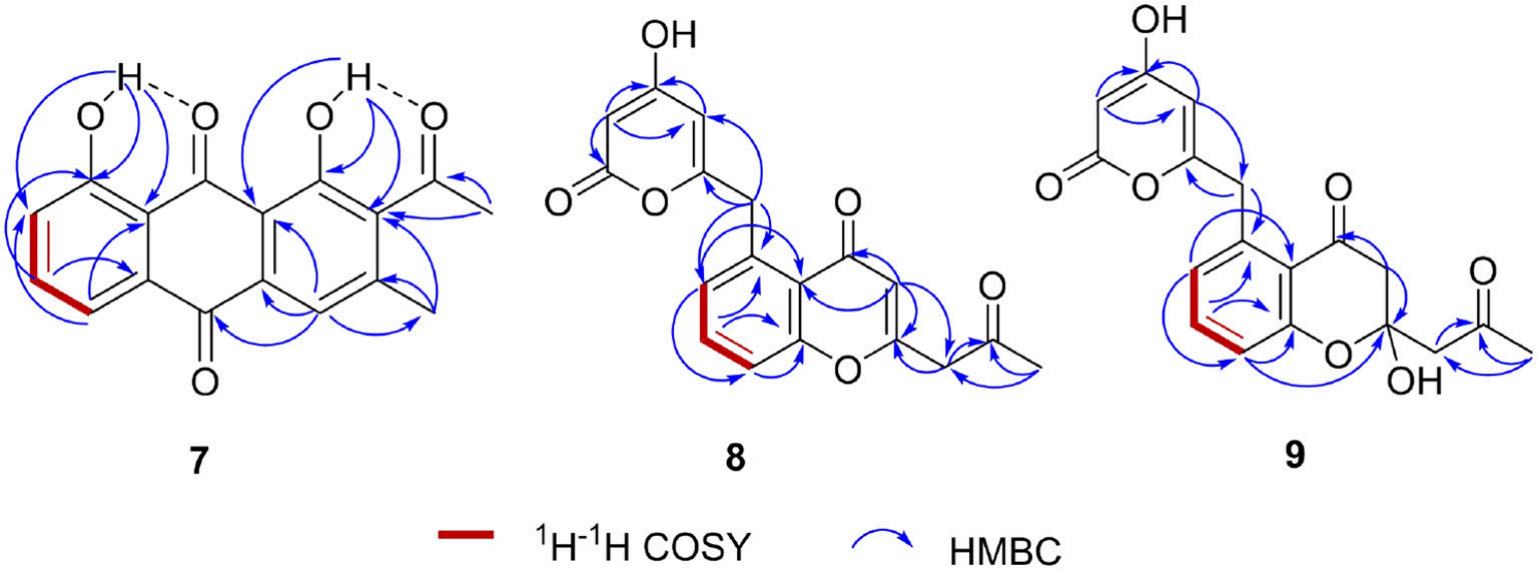
Key HMBC (→) and COZY (red bold bonds) correlations of huanglongmycin A-C (**7–9**).

HLM A (**7**) was obtained as a yellowish-brown solid. The molecular formula of **7** was determined as C_17_H_12_O_5_ based on HR-ESI-MS analysis, affording an [M - H]^−^ ion at *m/z* 295.0619 (calculated for [M - H]^−^ ion at *m*/*z* 295.0606), which indicated twelve degrees of unsaturation (Figure S8). Analysis of the ^1^H NMR, ^13^C NMR, HSQC, HMBC, and ^1^H-^1^H COZY spectra suggested that the structure of **7** was similar to several known anthraquinones DMAC (**5**) and SEK26 (**6**) from rationally designed type II PKSs (Figure 1, Figures S11–S15) (McDaniel et al., 1993, 1995). The presence of an acetyl group was based on the HMBC from H-12 (δ_H_ 2.55, -CH_3_) to C-11 (δ_C_ 202.8). The observation of HMBC from H-4 (δ_H_ 7.60) to C-4a (δ_C_ 133.1), C-9a (δ_C_ 114.1), C-3 (δ_C_ 145.6), and C-13 (δ_C_ 20.3), established the methyl group at C-3, hence the unambiguous assignment of the structure of **7** (Figure S14). Compound **7** was firstly obtained in the process to elucidate the structure of an anthraquinone protetrone through chemical degration, and later it was obtained in a medicinal chemistry program and also isolated from a *Streptomyces* strain, which was discovered from a soil sample in Mansoura, Egypt, but no detailed biological characterization was reported (McCormick and Jensen, 1968; Tietze et al., 2007; Abdelfattah, 2009). Compound **7** isolated from *S*. sp. CB9001 in this study showed consistent MS and NMR data with the previous reports.

HLM B (**8**) was obtained as a light yellow needle crystal. HR-ESI-MS data established the molecular formula of **8** as C_18_H_14_O_6_ (*m*/z 349.0687 [M + Na]^+^, calculated for [M + Na]^+^ ion at *m/z* 349.0687), indicating twelve degrees of unsaturation (Figure S9). The ^1^H NMR spectroscopic data of **8** revealed three aromatic protons at H-8 (δ_H_ 7.29, 1H, d, *J* = 7.4 Hz), H-9 (δ_H_ 7.71 dd, *J* = 7.4, 8.3 Hz), H-10 (δ_H_ 7.5, d, *J* = 8.3 Hz), which can be grouped into one ABX spin system with the aid of the COZY correlations of H-8/H-9/H-10 (Figure 2, Figures S16, S20). Further analysis of the NMR spectra suggested that the structure of **8** was similar to the known polyketide phaeochromycin C (**4**), except the presence of one additional keto carbonyl group C-17 (δ_C_ 202.7) (Figure 1, Figure S17) (Graziani et al., 2005). Presence of the pyrone moiety in **8** was confirmed by analyzing its proton and carbon NMR spectroscopic data, combined with the correlations of H-2 (δ_H_ 4.76) to C-1 (δ_C_ 163.8), C-3 (δ_C_ 170.5), and C-4 (δ_C_ 99.5), and H-4 (δ_H_ 5.25) to C-2 (δ_C_ 88.3), C-3 (δ_C_ 170.5), C-5 (δ_C_ 165.9), and C-6 (δ_C_ 37.4) in the HMBC spectrum (Figures S16–S19).

HLM C (**9**) was isolated as a light yellow solid. The molecular formula C_18_H_16_O_7_, was established upon analysis of the HR-ESI-MS peak at m/z 327.0870 [M - H_2_O + H]^+^ and 689.1872 [2M + H]^+^ (calculated for [M - H_2_O + H]^+^ ion at m/z 327.0869, and [2M + H]^+^ ion at m/z 689.1870), which afforded eleven degrees of unsaturation (Figure S10). The ^1^H, ^13^C and 2D NMR data obtained for HLM C (**9**) were similar to those obtained for **8**, except the presence of a methylene C-14 (δ_C_ 37.4), and a quaternary carbon C-15 (δ_C_ 100.5) connected to a hydroxy group (Table 1, Figures S21–S25). Its structure was further supported by the HMBC of H-14 (δ_H_ 47.9) to C-12 (δ_C_ 118.9), C-13 (δ_C_ 193.0), C-15 (δ_C_ 100.5), and C-16 (δ_C_ 52.4) (Figure S24).

### Identification of the putative biosynthetic gene cluster of huanglongmycins

**Figure 3:**
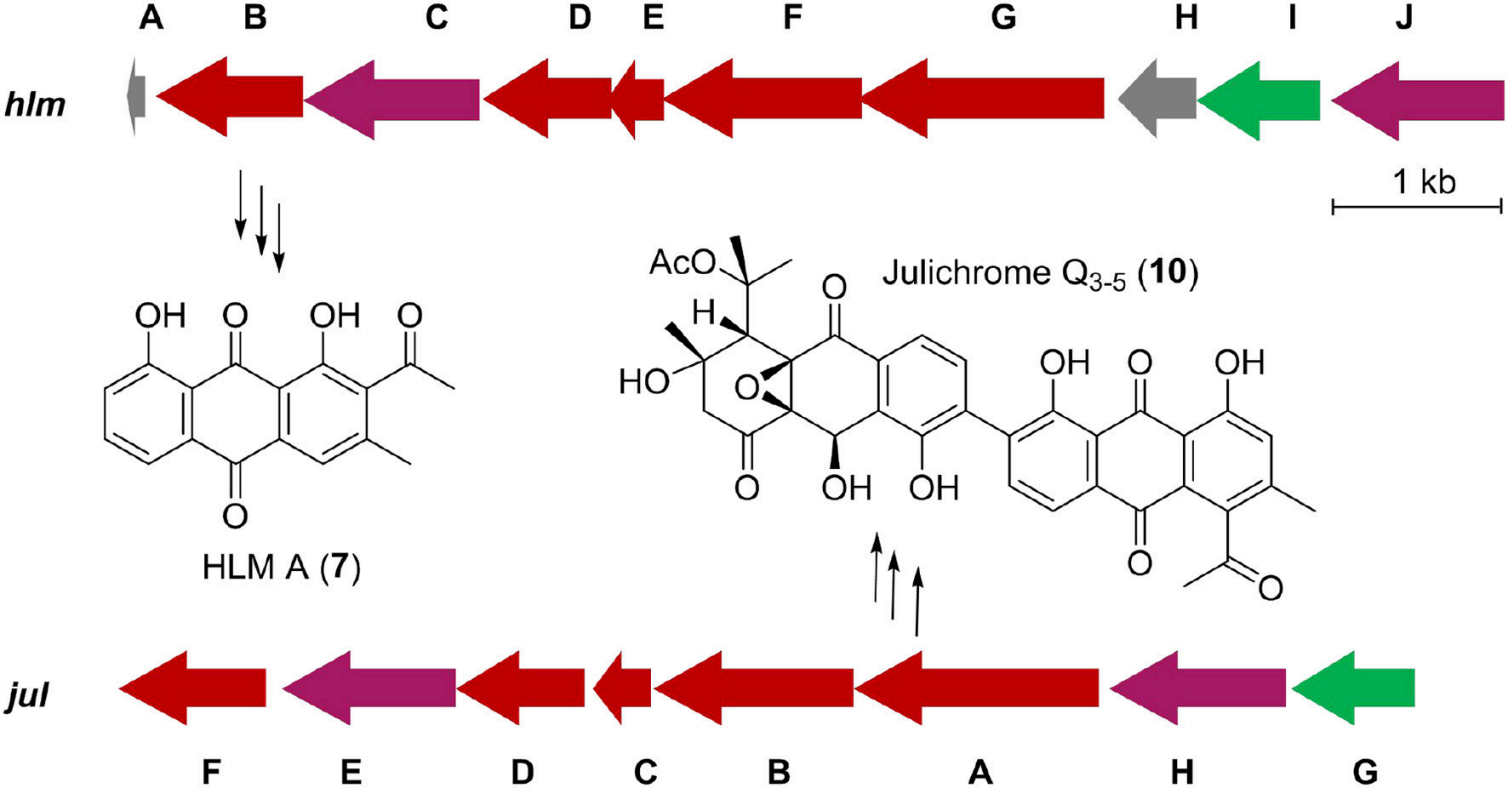
Predicted gene organization of huanglongmycin (*hlm*) cluster and julichrome (*jul*) cluster. Genes are color-coded according to their proposed functions. Red, purple, green, and gray represent core biosynthetic genes, additional biosynthetic genes, regulatory genes and other genes, respectively.

**Figure 4:**
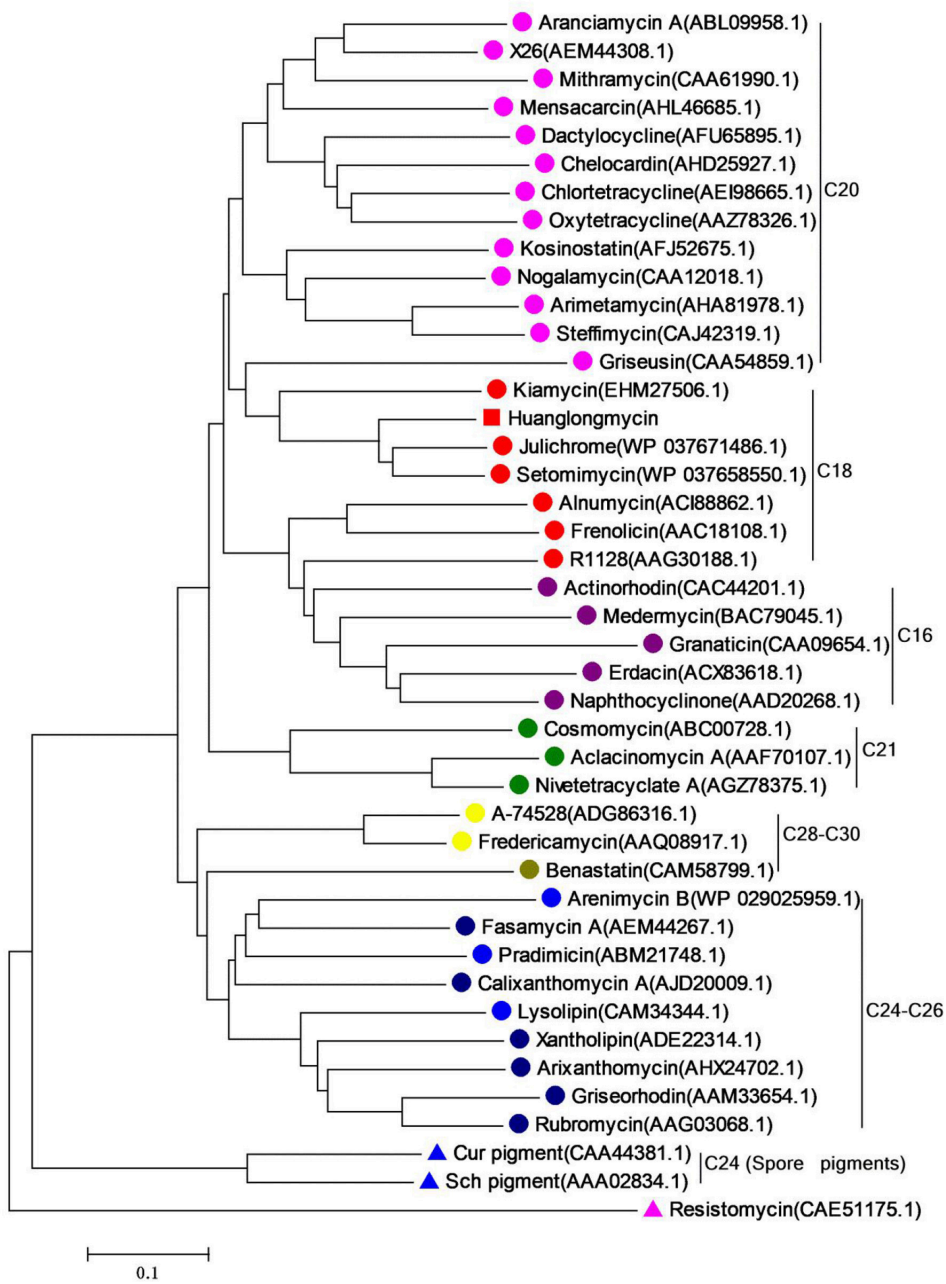
Phylogenetic analysis of the KS_β_ gene of huanglongmycin gene cluster. The same color circles (or triangles) represent the same polyketide chain length (noted on the right).

Inspired by the previous combinatorial biosynthesis approaches to generate the “unnatural” natural products by type II PKSs, such as **5** and **6**, and the fact that only a few biosynthetic gene clusters of nonaketides have been reported, we decided to identify the huanglongmycin (*hlm)* gene cluster in *S*. sp. CB09001 to shed light on the biosynthesis of HLM A–C (Figure 3, Table 2). The genome of *S*. sp. CB09001 was sequenced and bioinformatic analysis using antiSMASH revealed that there are three putative type II PKS gene clusters (GenBank accession number CP026730) (Blin et al., 2017). One gene cluster is likely to be involved in the biosynthesis of HLMs, and the other two share very high sequence homology to gray pigment or angucycline biosynthesis (Tables S1, S2). The putative KS_β_ gene *hlmF* is located in the KS_β_ group for nonaketide biosynthesis, based on the phylogenetic analysis of known 42 type II PKS gene clusters whose secondary metabolite products have been structurally characterized (Figure 4) (Hillenmeyer et al., 2015). Pairwise comparison of the annotated proteins between the *hlm* cluster with the julichrome biosynthetic gene cluster revealed high amino acid identity, strongly supporting that the *hlm* gene cluster most likely encodes the biosynthesis of HLMs in *S*. sp. CB09001 (Table 2).

**Table 2:** Alignment of the homologous proteins from the huanglongmycin (*hlm*) and the julichrome (*jul*) biosynthetic gene cluster.

**Gene**	**Deduced function of the encoded protein**	**aa**	**% identity/ % similarity**
HlmB/JuF	Thioesterase	311	67/77
HlmC/JulE	Cyclase	318	76/84
HlmD/JulD	ketoacyl reductase	262	87/94
HlmE/JulC	acyl carrier protein	88	75/89
HlmF/JulB	KS_β_	397	85/89
HlmG/JulA	KS_α_	443	89/94
HlmI/JulG	Transcriptional regulator	234	35/57
HlmJ/JulH	Cyclase	308	24/37

**Figure 5:**
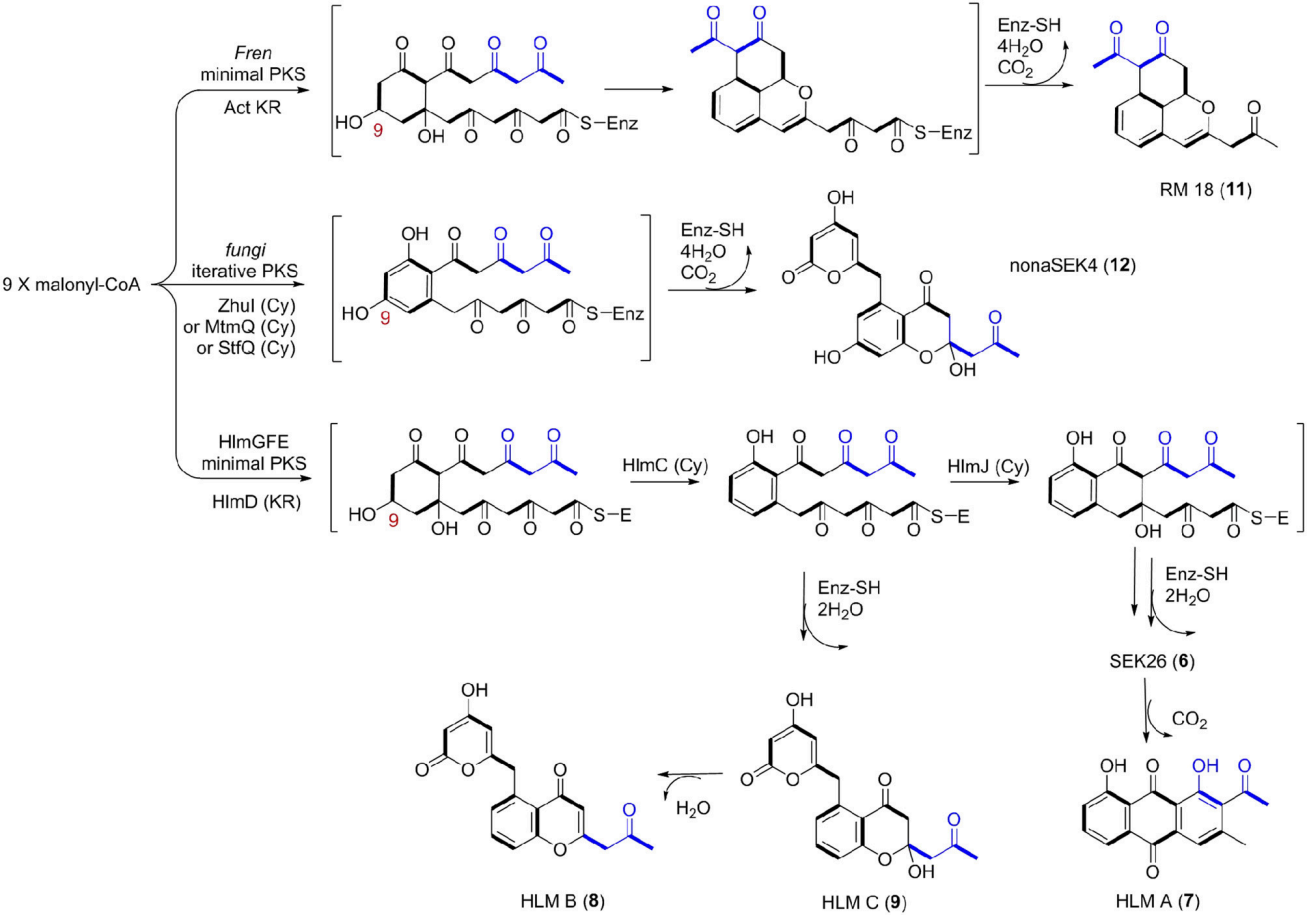
Biosynthetic Pathways for 18-Carbon Aromatic Polyketides. Biosynthesis between the known aromatic polyketides (i.e., nonaSEK4 and RM 18) and the putative HLM gene cluster were compared. The putative *hlm* minimal PKS contains HlmG, HlmF, and HlmE, the *Fren* PKS is the minimal PKS of frenolicin, while act KR is the ketoacyl reductase from actinorhodin PKS. The fungal iterative PKS is the megasynthase PKS4 from *Gibberella fujikuroi*. ZhuI, MtmQ, and StfQ are the cyclases of aromatic polyketide R1128, mithramycin, and steffimycin, respectively, which catalyzed the region-specific cyclization at C7–C12.

Thus, we now propose a pathway for HLM A–C biosynthesis, featuring a nonaketide intermediate by the HLM minimal PKS, containing KS_α_ (HlmG), KS_β_ (HlmF), and ACP (HlmE), which catalyzes the decarboxylative condensation of nine molecules of malonyl-CoA (Figure 5). Reduction of the poly-β-keto intermediate by the C-9 specific KR (HlmD), followed by the di-domain BexL-type reducing cyclase HlmC might afford the initial C7-C12 cyclization (Caldara-Festin et al., 2015). Interestingly, the isolation of the shunt metabolites HLM B and C suggested the leakage of the HLM polyketide intermediate during its transfer to the cyclase HlmJ under specific fermentation conditions, which were often observed in the engineered mutants or *in vitro* enzymatic assays (Shen and Hutchinson, 1993; Kharel et al., 2010). The formation of HLM A might be from the previous SEK26 (**6**) polyketide through decarboxylation, the MS of which was also observed in the fermentation extract from an *E. coli* strain containing a fungi PKS, one KR and two cyclases (Zhang et al., 2008). It was similar to the formation of anthraquione aloesaponarin II from DMAC (McDaniel et al., 1993). The above proposal was in agreement with the previous engineered biosynthesis of aromatic polyketide RM 18 (**11**) and nonaSEK14 (**12**) from the same nonaketide intermediate, albeit from a wild-type *Streptomyces* strain (Figure 5) (McDaniel et al., 1993; Hopwood, 1997; Zhang et al., 2008). The similar biosynthetic pathway for huanglongmycin A might be also operational in the *Streptomyces* strain isolated from Egypt, which suggested that natural nonaketides might be underappreciated (Abdelfattah, 2009).

### The biological evaluation of huanglongmycins

HLM A–C (**7–9**) were first evaluated for their antibacterial activities against *Staphylococcus aureus* ATCC 29213, and several clinical isolates from local hospitals, including methicillin-sensitive *S. aureus* (MSSA) strain and methicillin-resistant *S. aureus* (MRSA) strains, as well as strains of *Escherichia coli, Klebsiella pneumoniae* and *Pseudomonas aeruginosa*, using chloramphenicol and linezolid as controls (Table 3). HLM A showed weak antibacterial activities against the gram-negative pathogens *P. aeruginosa* and *E. coli*, with minimum inhibitory concentrations (MICs) of 64 μg/mL. HLM B showed weak antibacterial activities against the tested *S. aureus* strains, with a MIC of 64 μg/mL, while HLM C showed no antibacterial activities against the five indicator strains (Table 3).

**Table 3:** Antibacterial activities (MICs, μg/mL) and cytotoxic activities (IC_50_, μM) of huanglongmycin A–C (**7–9**). *S. aureus* 29213, *Staphylococcus aureus* ATCC 29213; MSSA, Methicillin-sensitive *Staphylo-coccus aureus*; MRSA, Methicillin-resistant *Staphylococcus aureus*; –, not tested.

**Bioactivity**	**Bacteria/cancer cells**	**Compounds**					
		**Linezolid**	**Chloramphenicol**	**Mitomycin**	**HLM A (7)**	**HLM B (8)**	**HLM C (9)**
Antibacterial activities	*S. aureus* ATCC 29213	1	2	–	>64	64	>64
	MSSA	1	2	–	>64	64	>64
	MRSA	1	2	–	>64	64	>64
	*E. coli*	>64	8	–	64	>64	>64
	*K. pneumoniae*	>64	8	–	>64	>64	>64
	*P. aeruginosa*	>64	>64	–	64	>64	>64
Cytotoxicity	A549	–	–	0.9 ± 0.1	13.8 ± 1.5	>60	>60
	SKOV3	–	–	–	41.3 ± 7.0	>60	>60
	Hela	–	–	–	43.7 ± 2.3	>60	>60
	Caco-2	–	–	–	43.2 ± 2.1	>60	>60

The *in vitro* cytotoxicity of compounds **7**–**9** were evaluated against four human cancer cell lines, including non-small cell lung cancer cell line A549, epithelial cancer cell line SKOV3, Hela and human epithelial colorectal adenocarcinoma (Caco-2) using MTT assay. HLM A showed moderate cytotoxicity against A549 (IC_50_ = 13.8 ± 1.5 μM) and weak cytotoxicities against SKOV3, Hela and Caco-2 (IC_50_ = 41.3 ± 7.0, 43.7 ± 2.3, and 43.2 ± 2.1 μM), while HLM B and C showed no cytotoxicities at the tested condition (IC_50_ > 60 μM).

## Conclusion

In summary, this work describes the isolation and characterization of three natural products HLM A-C, and the identification of a putative gene cluster, featuring a type II PKS specific for nonaketide biosynthesis from *S*. sp. CB09001. The proposal for HLMs biosynthesis agreed well with the previous engineered biosynthesis for bacteria aromatic polyketides in the past few decades (McDaniel et al., 1993, 1995; Hopwood, 1997; Kramer et al., 1997; Yu et al., 1998; Shen, 2000; Hertweck et al., 2007; Zhang et al., 2008, 2017; Das and Khosla, 2009; Zhou et al., 2010). Albeit previously isolated, HLM A was reported for the first time, to exhibit moderate cytotoxicity against A549 cancer cell line (IC_50_ = 13.8 ± 1.5 μM) and weak antibacterial activity against *P. aeruginosa* (MICs = 64 μg/mL). This study showcased once again Nature is the ultimate combinatorial biosynthetic chemist, and should inspire continued efforts for natural product discovery from unexplored and underexplored ecological niches.

## Author contributions

YH, YD, and BS: conceived the project; LJ and HP: performed experiments; JX and MS: carried out the cytotoxicity assays; XY, DY, and XZ: contributed to bioinformatics analysis; YH and LJ: wrote the manuscript with help from the other authors.

### Conflict of interest statement

The authors declare that the research was conducted in the absence of any commercial or financial relationships that could be construed as a potential conflict of interest.
