# Supplementary material for: Huanglongmycin A-C, Cytotoxic Polyketides Biosynthesized by a Putative Type II Polyketide Synthase From Streptomyces sp. CB09001
Source: Front Chem. 2018 Jun 26;6:254. doi: 10.3389/fchem.2018.00254 (PMC6036704; doi:10.3389/fchem.2018.00254)

# Supporting Information

## Huanglongmycin A-C, Cytotoxic Polyketides Biosynthesized by a Putative Type II Polyketide Synthase from *Streptomyces* sp. CB09001

Lin Jiang,[†] Hong Pu,[†] Jingxi Xiang,[†] Meng Su,[†] Xiaohui Yan,[#] Dong Yang,[#] Xiangcheng Zhu,[†, ⊥] Ben Shen,[#, ¶, §] Yanwen Duan[*, †, ⊥, Δ] and Yong Huang[*, †, Δ]

[†]Xiangya International Academy of Translational Medicine at Central South University, Changsha, Hunan 410013, China;

[⊥]Hunan Engineering Research Center of Combinatorial Biosynthesis and Natural Product Drug Discovery，

[Δ]National Engineering Research Center of Combinatorial Biosynthesis for Drug Discovery, Changsha, Hunan 410013, China;

[#]Departments of Chemistry and [¶]Molecular Medicine, and [§]Natural Products Library Initiative at The Scripps Research Institute, The Scripps Research Institute, Jupiter, FL 33458, USA.

*To whom correspondents should be addressed: Yong Huang, Central South University, Tongzipo Road，#172，Yuelu District，Changsha, Hunan 410013，China. Tel：（86）731 8265 0539；Fax：（86）731 8265 0551； Email：jonghuang@csu.edu.cn or Yanwen Duan, Email: ywduan66@sina.com

# Table of Contents

**Table S1** Predicted functions of ORFs in the huanglongmycin gene cluster. The gene cluster may contain 21 ORFs including HlmA to HlmU. ORF (-1) to ORF (-6) and ORF (+1) to ORF (+8) are genes beyond the putative boundaries......3
**Table S2** Similarity of the huanglongmycin, frenolicin and julichrome gene clusters......4
**Figure S1** HPLC analysis of fermentation broth of *Streptomyces* sp. CB09001 in 50 mL G1 fermentation medium supplemented with triclosan......5
**Figure S2** HPLC analyses of fermentation broth of *Streptomyces* sp. CB09001 in G1 fermentation medium with different fermentation volume......6
**Figure S3** UPLC-MS analyses of fermentation broth of *Streptomyces* sp. CB09001 fermented in 50 mL standard G1 fermentation medium added with 3% macroporous resin DA201-H......7
**Figure S4** UV spectrum of HLM A (**7**) (A), HLM B (**8**) (B), and HLM C (**9**) (C)......8
**Figure S5** CD spectrum of HLM C (**9**) in DMSO......9
**Figure S6** IR spectrum of HLM B (**8**)......10
**Figure S7** IR spectrum of HLM C (**9**)......10
**Figure S8** HRESIMS spectrum of HLM A (**7**)......11
**Figure S9** HRESIMS spectrum of HLM B (**8**)......11
**Figure S10** HRESIMS spectrum of HLM C (**9**)......11
**Figure S11** $^{1}H$ NMR spectrum of HLM A (**7**) in $CDCl_3$ (500 MHz)......12
**Figure S12** $^{13}C$ NMR spectrum of HLM A (**7**) in $CDCl_3$ (125 MHZ)......13
**Figure S13** HSQC spectrum of HLM A (**7**)......14
**Figure S14** HMBC spectrum of HLM A (**7**)......15
**Figure S15** $^{1}H$-$^{1}H$ COSY spectrum of HLM A (**7**)......16
**Figure S16** $^{1}H$ NMR spectrum of HLM B (**8**) in DMSO-$d_6$ (500 MHz)......17
**Figure S17** $^{13}C$ NMR spectrum of HLM B (**8**) in DMSO-$d_6$ (125 MHZ)......18
**Figure S18** HSQC spectrum of HLM B (**8**)......19
**Figure S19** HMBC spectrum of HLM B (**8**)......20
**Figure S20** $^{1}H$-$^{1}H$ COSY spectrum of HLM B (**8**)......21
**Figure S21** $^{1}H$ NMR spectrum of HLM C (**9**) in DMSO-$d_6$ (500 MHz)......22
**Figure S22** $^{13}C$ NMR spectrum of HLM C (**9**) in DMSO-$d_6$ (125 MHZ)......23
**Figure S23** HSQC spectrum of HLM C (**9**)......24
**Figure S24** HMBC spectrum of HLM C (**9**)......25
**Figure S25** $^{1}H$-$^{1}H$ COSY spectrum of HLM C (**9**)......26

**Table S1** Predicted functions of ORFs in the huanglongmycin gene cluster. The gene cluster may contain 21 ORFs including HlmA to HlmU. ORF (-1) to ORF (-6) and ORF (+1) to ORF (+8) are genes beyond the putative boundaries.

| Gene | aa | Protein homologue (Origin) | NCBI accession no. | %ID/ %SI |
|---|---|---|---|---|
| ORF (-6) | 198 | histidine phosphatase [*S.* sp. WM6391] | KKD13693.1 | 96/96 |
| ORF (-5) | 329 | membrane protein [*S.* sp. WM6391] | KKD13692.1 | 93/96 |
| ORF (-4) | 360 | hypothetical protein TR66_19350 [*S.* sp. WM6391] | KKD13742.1 | 92/93 |
| ORF (-3) | 557 | membrane protein [*S.* sp. WM6391] | KKD13691.1 | 94/94 |
| ORF (-2) | 453 | helicase [*S.* sp. WM6391] | KKD13690.1 | 98/98 |
| ORF (-1) | 51 | unknown | \ | \ |
| HlmA | 58 | hypothetical protein AQJ54_24600 [*S. griseorubiginosus*] | KUN64213.1 | 83/88 |
| HlmB | 311 | thioesterase [*S. griseorubiginosus*] | KUN64211.1 | 69/77 |
| HlmC | 318 | cyclase [*S. griseorubiginosus*] | KUN64210.1 | 73/83 |
| HlmD | 262 | ketoacyl reductase [*S. griseorubiginosus*] | KUN64209.1 | 88/96 |
| HlmE | 88 | acyl carrier protein [*S. griseorubiginosus*] | KUN64208.1 | 83/93 |
| HlmF | 397 | beta-ketoacyl synthase *[S. griseorubiginosus*] | KUN64207.1 | 84/89 |
| HlmG | 443 | beta-ACP synthase [*S. griseorubiginosus*] | KUN64206.1 | 88/93 |
| HlmH | 154 | hypothetical protein CW362_05350 [*S.* sp. A249] | PKT74077.1 | 75/82 |
| HlmI | 234 | TetR/AcrR family transcriptional regulator [*S.* sp. A249] | PKT74078.1 | 59/74 |
| HlmJ | 308 | cyclase [*S.* sp. DvalAA-14] | SCD75377.1 | 56/67 |
| HlmK | 99 | antibiotic biosynthesis monooxygenase *[S.* sp. A249] | PKT74080.1 | 58/68 |
| HlmL | 273 | hypothetical protein CW362_05310 [*S.* sp. A249] | PKT74069.1 | 63/75 |
| HlmM | 482 | MFS transporter [*S.* sp. A249] | PKT74081.1 | 69/78 |
| HlmN | 211 | MarR family transcriptional regulator [*S.* sp. A249] | PKT74082.1 | 52/65 |
| HlmO | 298 | MBL metalloprotease [*S.* sp. A249] | PKT74133.1 | 62/74 |
| HlmP | 554 | long-chain acyl-CoA synthetase [*S.* sp. 1] | PIG59384.1 | 45/58 |
| HlmQ | 165 | hydroxylacyl-CoA dehydrogenase [*S. swartbergensis*] | OUD04976.1 | 49/57 |
| HlmR | 156 | bifunctional aromatase (cyclase/dehydratase) [*S.* sp. KS_5] | SEE83731.1 | 36/51 |
| HlmS | 198 | TetR family transcriptional regulator [*S.* sp. NRRL F-6602] | KPC90247.1 | 89/92 |
| HlmT | 294 | NmrA family transcriptional regulator [*S.* sp. CB02414] | OKI84162.1 | 83/91 |
| HlmU | 188 | deaminase [*S.* sp. M1013] | OMI89221.1 | 88/92 |
| ORF (+1) | 136 | ATP-binding protein [*S. pactum*] | AQS69439.1 | 93/97 |
| ORF (+2) | 283 | DNA-binding protein [*S. resistomycificus*] | KUN93067.1 | 75/86 |
| ORF (+3) | 66 | DUF397 domain-containing protein [*S. pactum*] | AOW91123.1 | 87/92 |
| ORF (+4) | 285 | methyltransferase [*S. coelicolor* A3(2)] | NP_627043.1 | 93/95 |
| ORF (+5) | 197 | TetR-family transcriptional regulator [*S. lividans* 1326] | EOY47876.1 | 93/94 |
| ORF (+6) | 366 | LLM class flavin-dependent oxidoreductase [*S.* sp. CS159] | OWA10306.1 | 98/99 |
| ORF (+7) | 603 | diguanylate cyclase/phosphodiesterase [*S.* sp. 2114.2] | SDT46921.1 | 97/97 |
| ORF (+8) | 420 | conserved hypothetical protein *[S. lividans* TK24] | EFD69070.1 | 97/97 |

**Table S2** Similarity of the huanglongmycin, frenolicin and julichrome gene clusters.

| Gene | Putative function of the encoded protein | aa | Protein homologue (NCBI accession no.) (Compound) | %ID/ %SI (query cover) |
|---|---|---|---|---|
| HlmA | hypothetical protein | 58 | / | - |
| HlmB | thioesterase | 311 | / | - |
| | | | WP_020275100.1 (julichrome) | 67/77 |
| HlmC | putative cyclase/ dehydrase | 318 | AAC18112.1(frenolicin) | 38/51 |
| | | | WP_020275099.1 (julichrome) | 76/84 |
| HlmD | ketoacyl reductase | 262 | AAC18111.1 (frenolicin) | 63/75 |
| | | | WP_020275098.1 (julichrome) | 87/94 |
| HlmE | acyl carrier protein | 88 | AAC18109.1) (frenolicin) | 53/68 |
| | | | WP_020275097.1 (julichrome) | 75/89 |
| HlmF | keto synthase beta | 397 | AAC18108.1 (frenolicin) | 58/70 |
| | | | WP_037671486.1 (julichrome) | 85/89 |
| HlmG | keto synthase alpha | 443 | AAC18107.1 (frenolicin) | 67/79 |
| | | | WP_020275095.1 (julichrome) | 89/94 |
| HlmH | hypothetical protein | 154 | / | - |
| HlmJ | cyclase | 308 | AAC18113.1 (frenolicin) | 50/64 |
| HlmK | antibiotic biosynthesis monooxygenase | 99 | AAC18116.1 (frenolicin) | 30/45 |
| HlmL | hypothetical protein | 273 | AAC18102.1 (frenolicin) | 40/56 |
| HlmM | MFS transporter | 482 | AAC18101.1 (frenolicin) | 39/56 |
| | | | WP_063610769.1 (julichrome) | 35/51 |
| HlmN | MarR family transcriptional regulator | 211 | / | - |
| HlmO | MBL metalloprotease | 298 | / | - |
| HlmP | long-chain acyl-CoA synthetase | 554 | / | - |
| HlmQ | hydroxylacyl-CoA dehydrogenase | 165 | AAC18110.1 (frenolicin) | 36/44 |
| HlmR | bifunctional aromatase | 156 | AAC18110.1 (frenolicin) | 31/38 |
| HlmS | TetR family transcriptional regulator | 198 | WP_037671481.1 (julichrome) | 32/44 |
| HlmT | NmrA family transcriptional regulator | 294 | AAC18114.1 (frenolicin) | 30/42 |
| | | | WP_020275086.1 (julichrome) | 31/42 |
| HlmU | deaminase | 188 | AAC18104.1 (frenolicin) | 30/37 |

**Figure S1** HPLC analysis of fermentation broth of *Streptomyces* sp. CB09001 in 50 mL G1 fermentation medium supplemented with triclosan in 250-mL flat-bottom Erlenmeyer flasks. Different concentration of triclosan (0 - 20 μM) was used: I, 0; II, 0.002 μM; III, 0.02 μM; IV, 0.2 μM; V, 2 μM; VI, 20 μM. ▼, HLM A (**7**); ▽, HLM C (**9**).

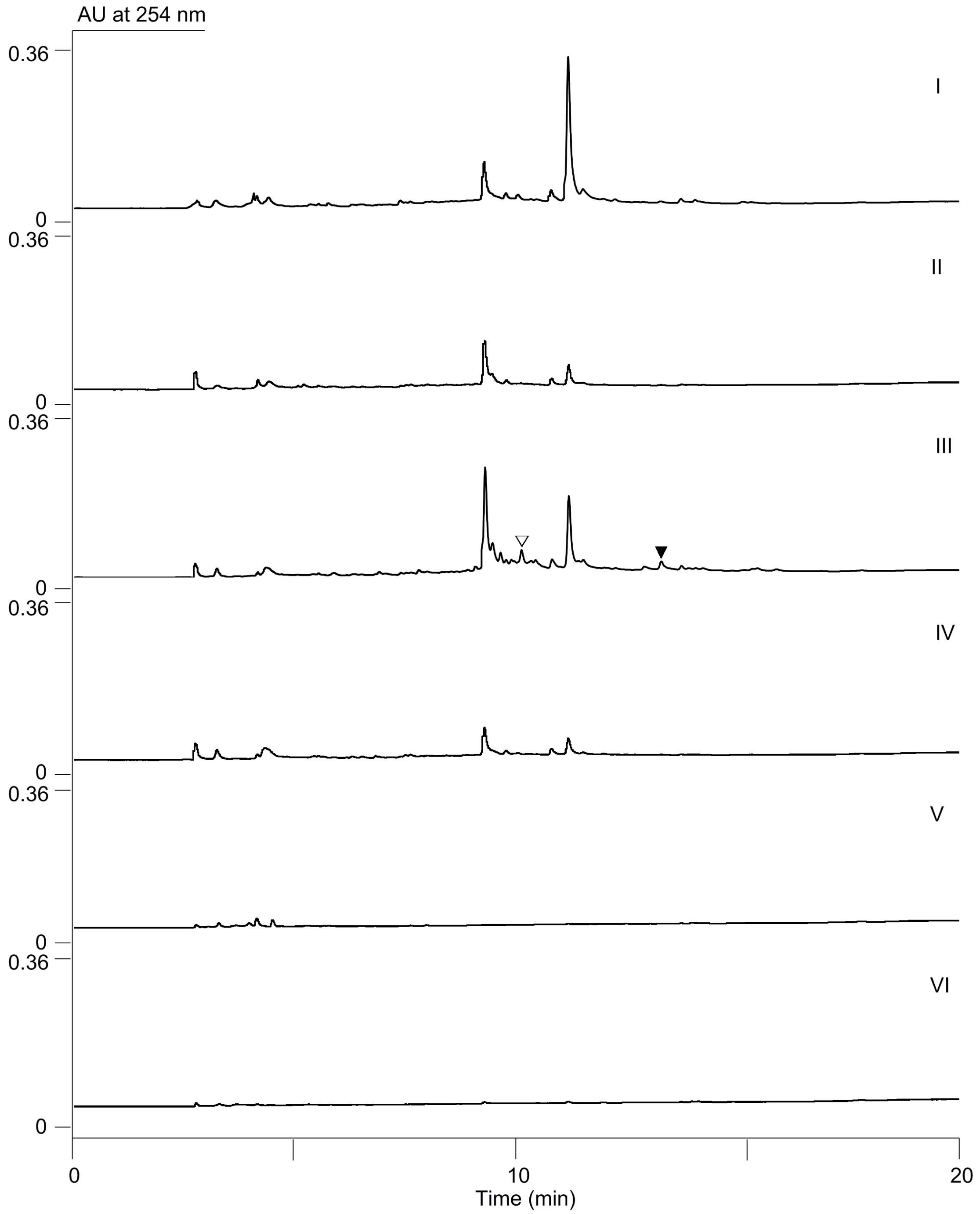

**Figure S2** HPLC analyses of fermentation broth of *Streptomyces* sp. CB09001 in G1 fermentation medium with different fermentation volume in 250-mL flat-bottom Erlenmeyer flasks from 25 mL to 150 mL per flask. I, 25 mL; II, 50 mL; III, 75 mL; IV, 100 mL; V, 125 mL; VI, 150 mL. ▼, HLM A (**7**); ◇, HLM B (**8**); ▽, HLM C (**9**).

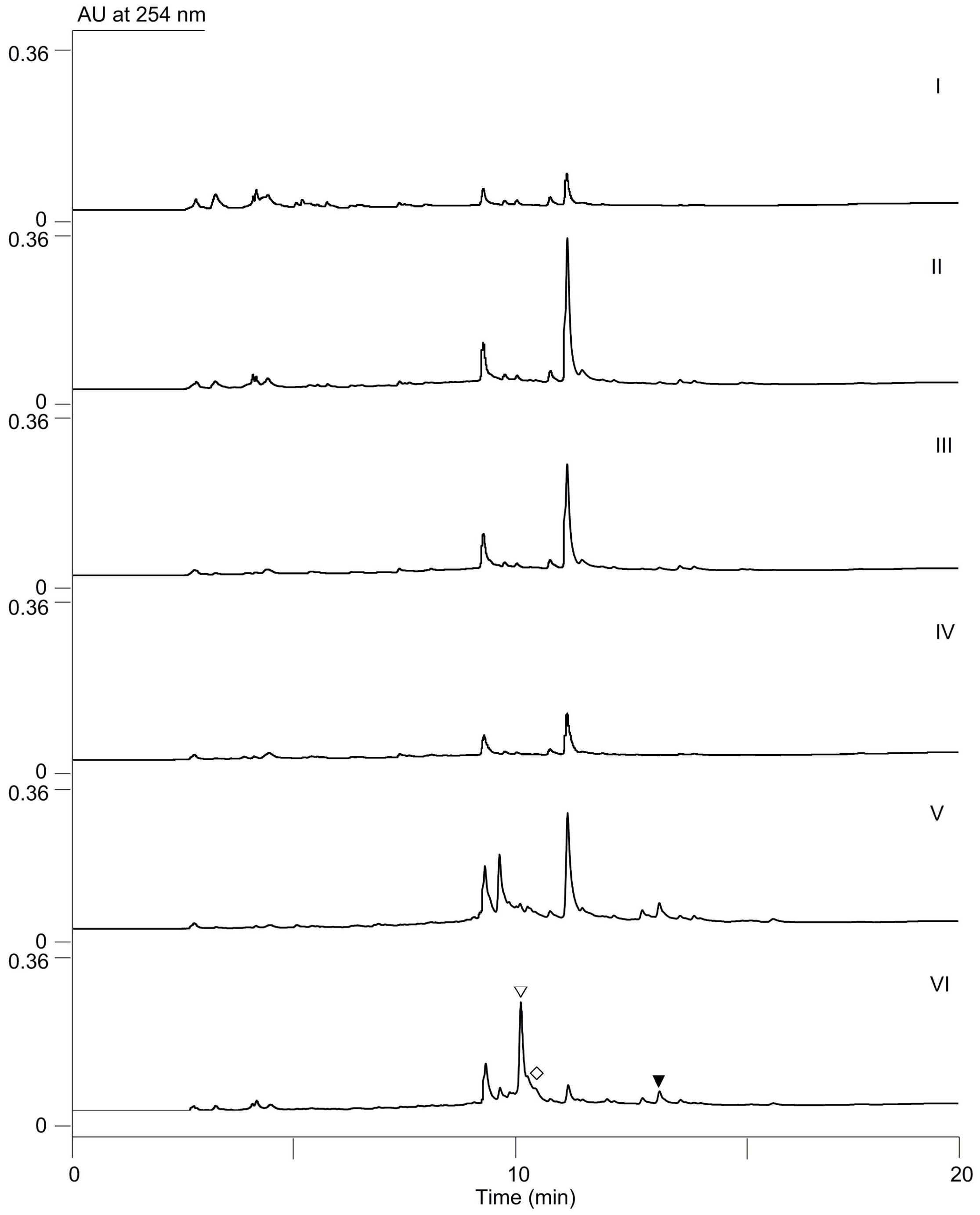

**Figure S3** UPLC-MS analyses of fermentation broth of *Streptomyces* sp. CB09001 fermented in 50 mL standard G1 fermentation medium added with 3% macroporous resin DA201-H in 250 mL Erlenmeyer flasks. (A) UPLC analyses of the fermentation profile. (B) (+)-ESIMS and (−)-ESIMS spectrum of HLM A (**7**). ▼, HLM A (**7**).

A

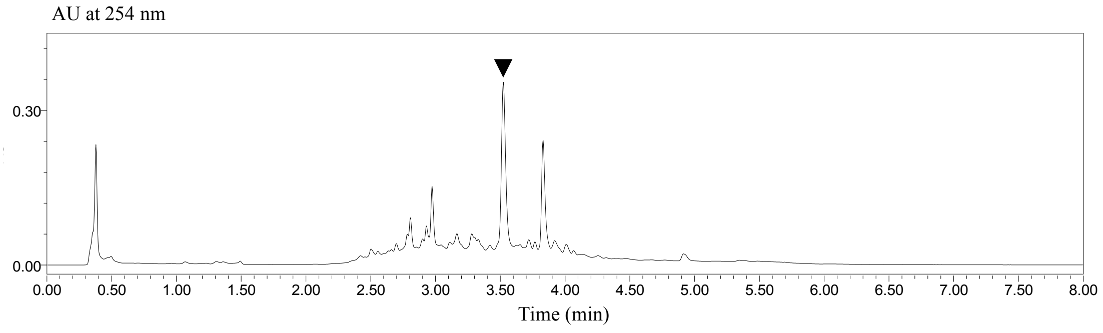


B

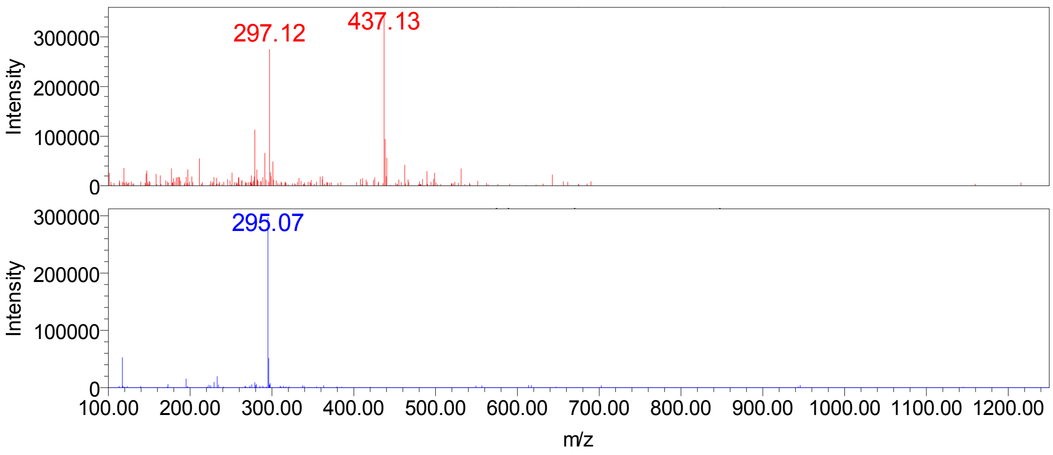

**Figure S4** UV spectrum of HLM A (**7**) (A), HLM B (**8**) (B), and HLM C (**9**) (C)

A

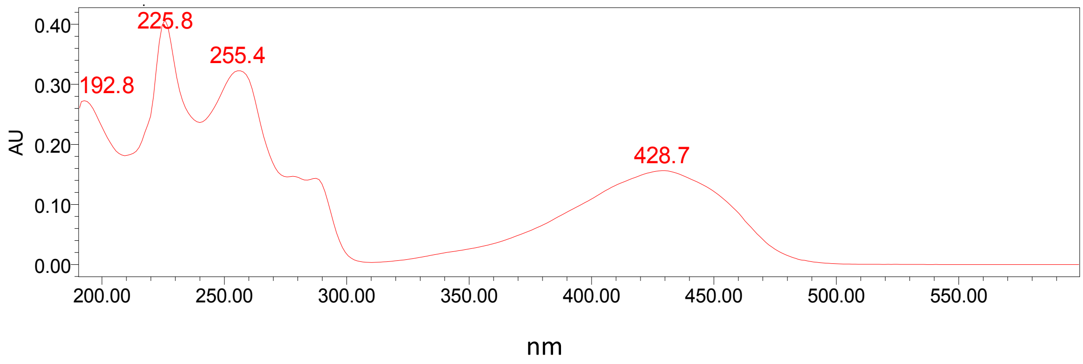


| No. | Wavelength (nm) | Abs |
|---|---|---|
| 1 | 192.8 | 0.268 |
| 2 | 225.8 | 0.388 |
| 3 | 255.4 | 0.300 |
| 4 | 428.7 | 0.144 |

B

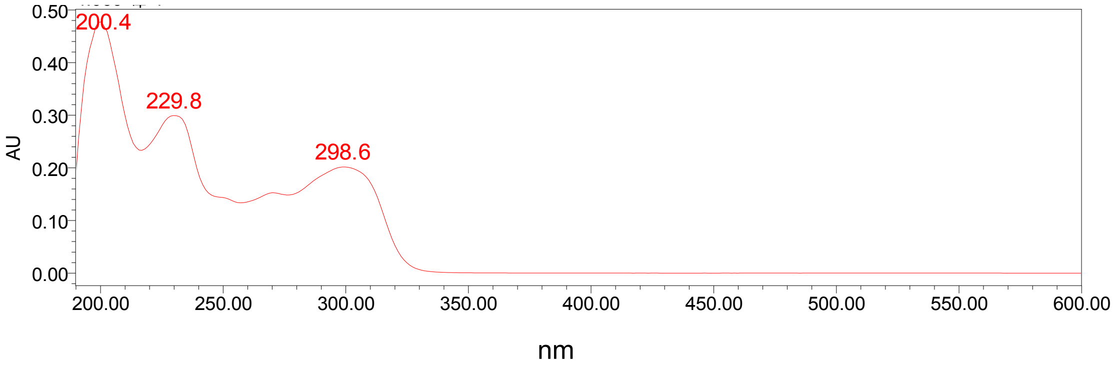


| No. | Wavelength (nm) | Abs |
|---|---|---|
| 1 | 200.4 | 0.473 |
| 2 | 229.8 | 0.297 |
| 3 | 298.6 | 0.200 |

C

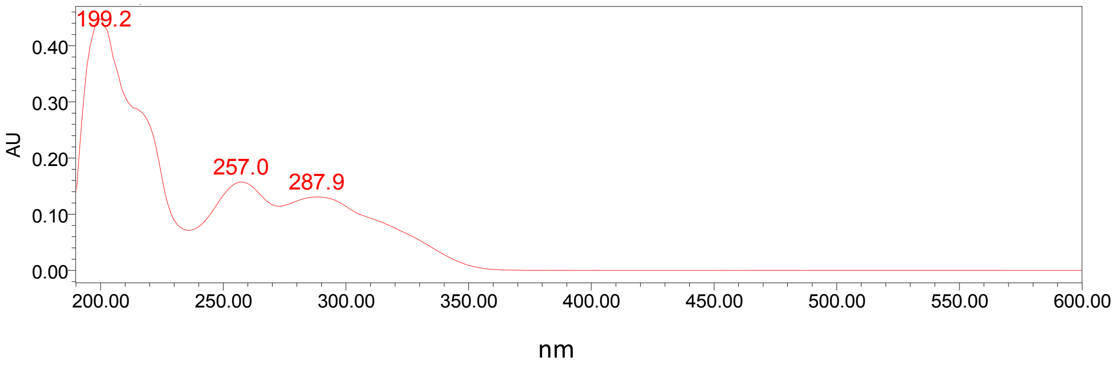


| No. | Wavelength (nm) | Abs |
|---|---|---|
| 1 | 199.2 | 0.488 |
| 2 | 257.0 | 0.170 |
| 3 | 287.9 | 0.142 |

**Figure S5** CD spectrum of HLM C (**9**) in DMSO

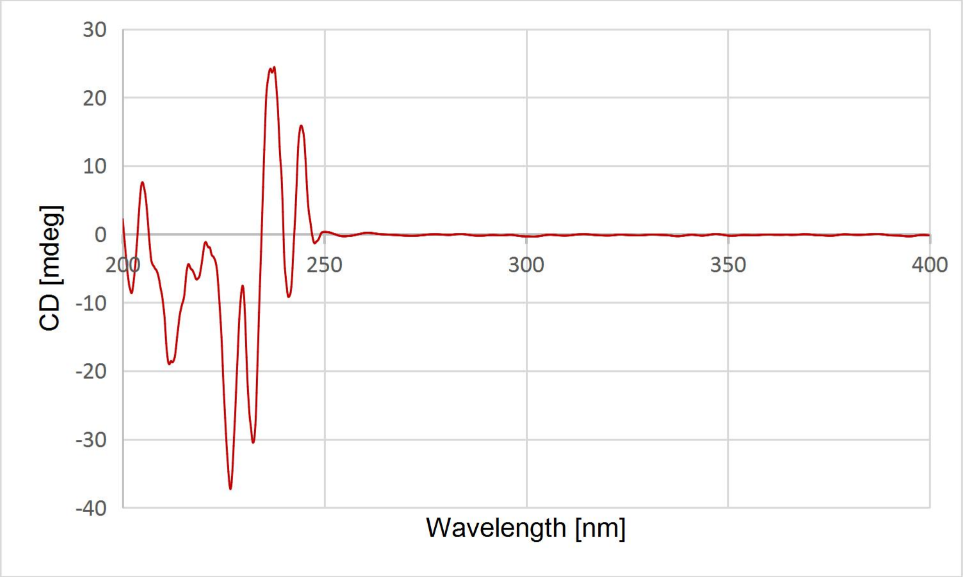

**Figure S6** IR spectrum of HLM B (**8**)

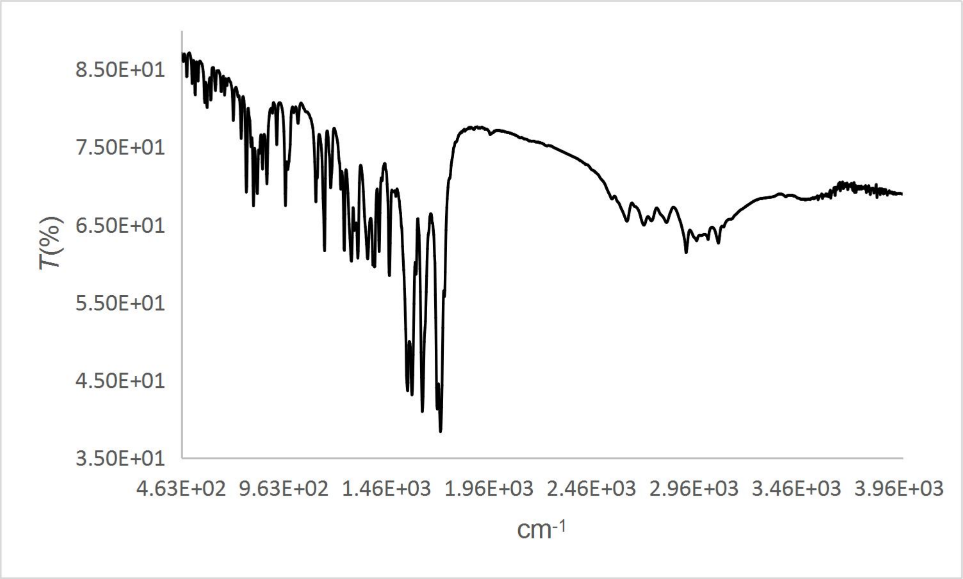


**Figure S7** IR spectrum of HLM C (**9**)

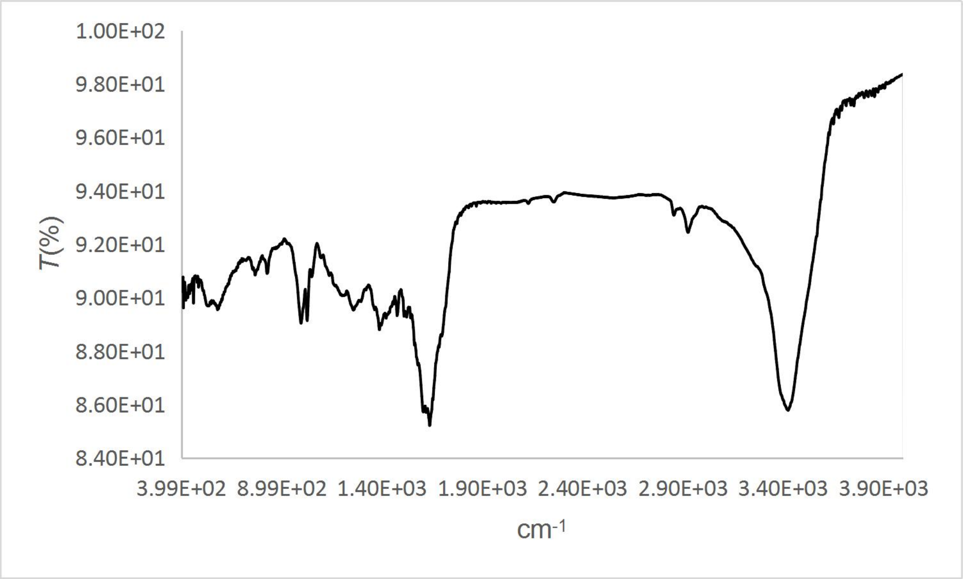

**Figure S8** HRESIMS spectrum of HLM A (**7**)

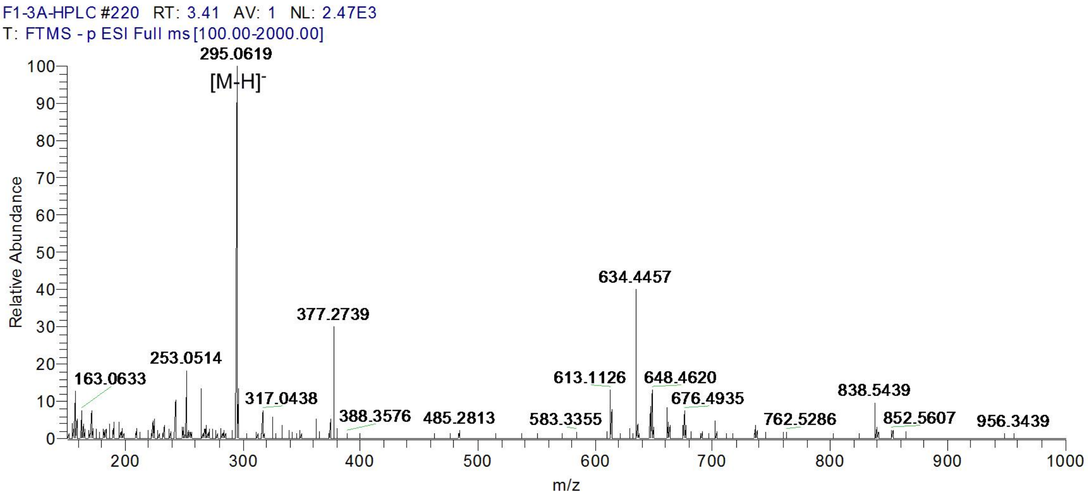


**Figure S9** HRESIMS spectrum of HLM B (**8**)

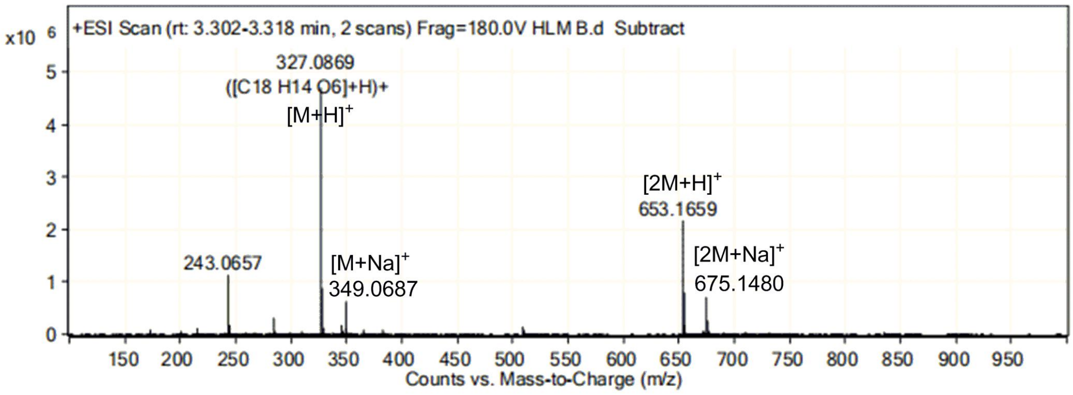


**Figure S10** HRESIMS spectrum of HLM C (**9**)

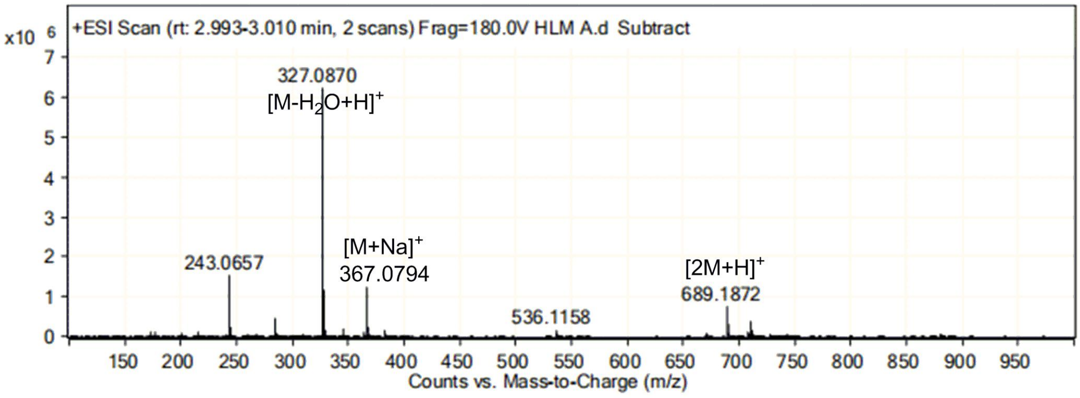

**Figure S11** $^{1}$H NMR spectrum of HLM A (**7**) in $CDCl_3$ (500 MHz)

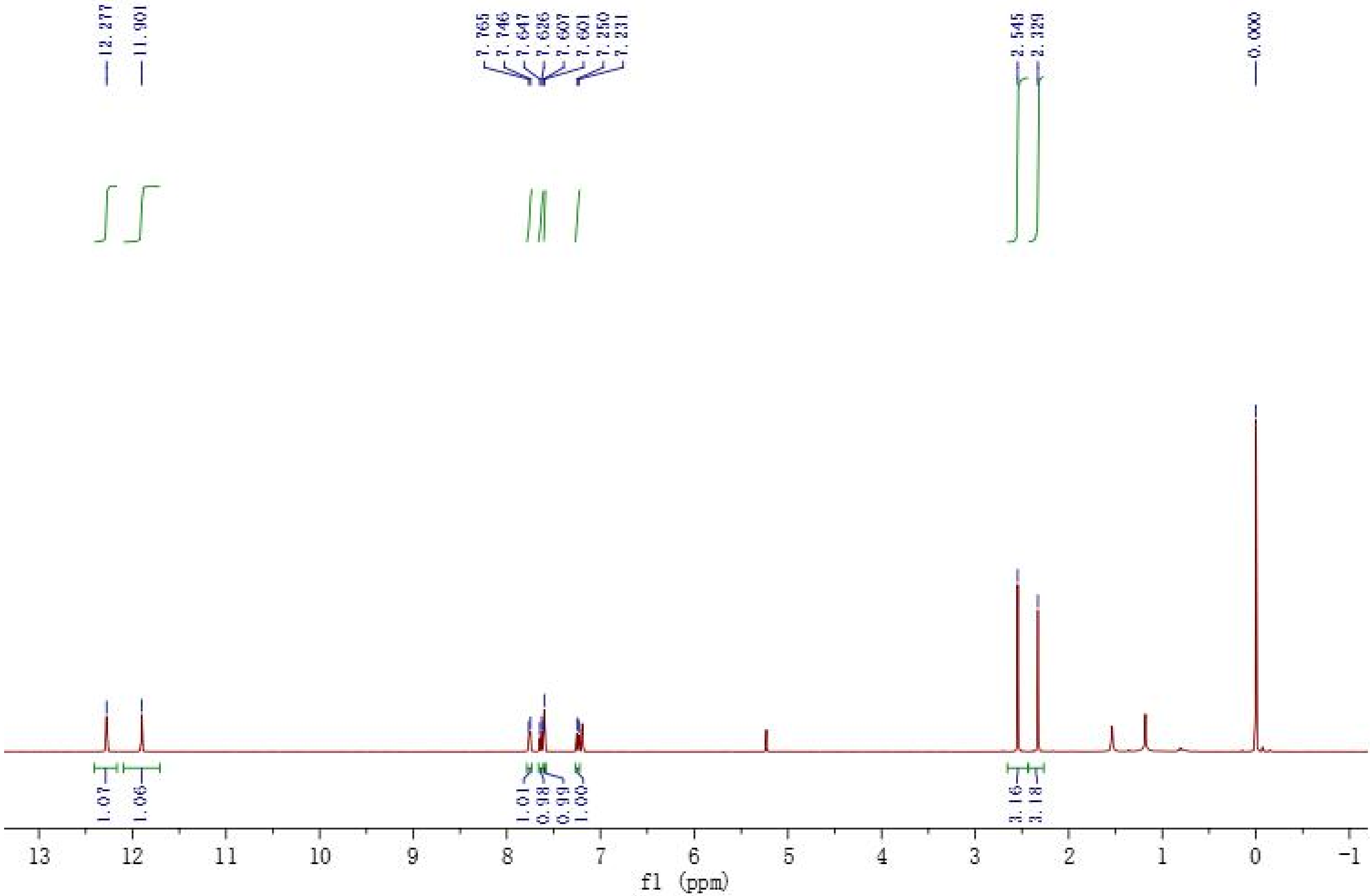

**Figure S12** $^{13}C$ NMR spectrum of HLM A (**7**) in $CDCl_3$ (125 MHz)

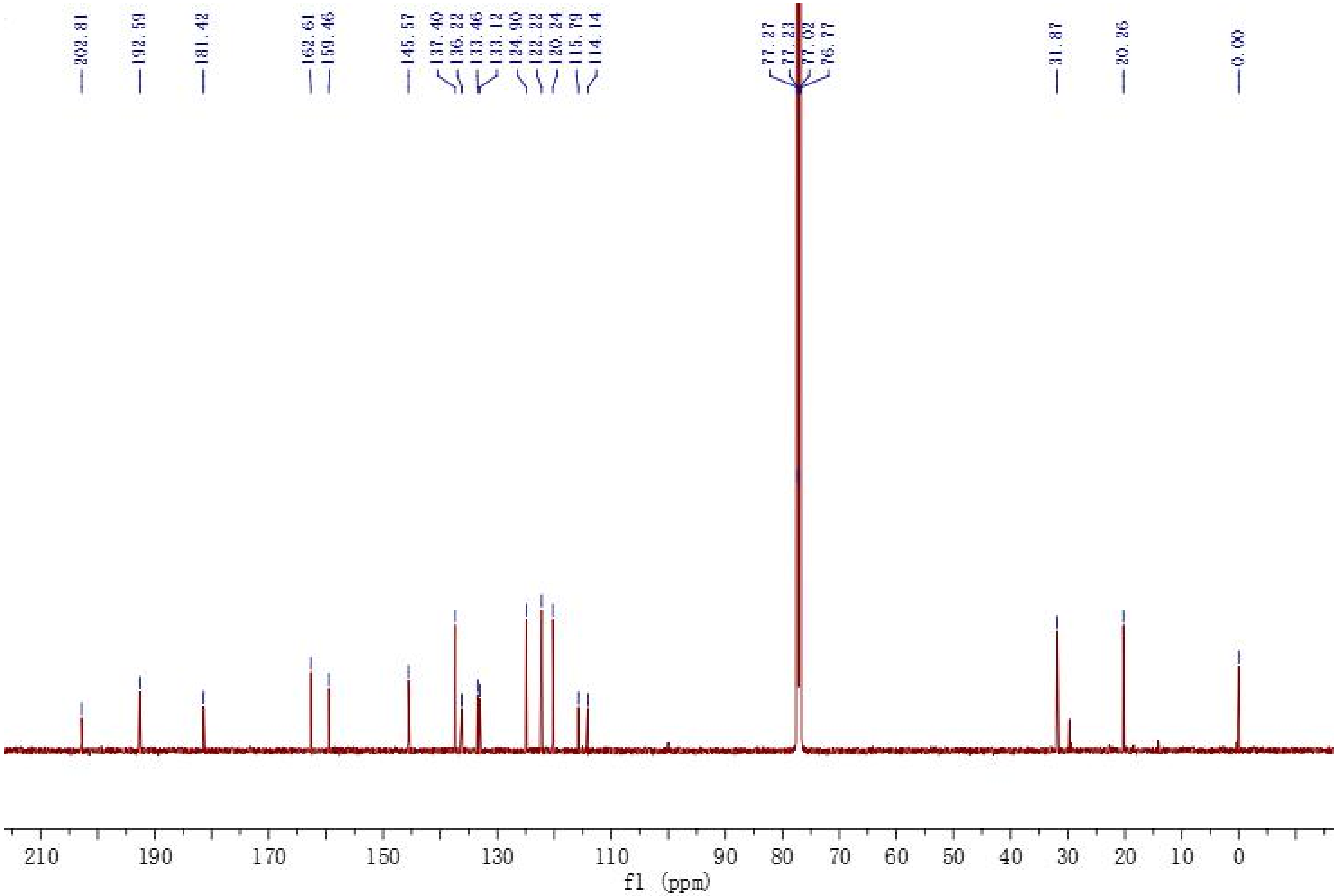

**Figure S13** HSQC spectrum of HLM A (**7**)

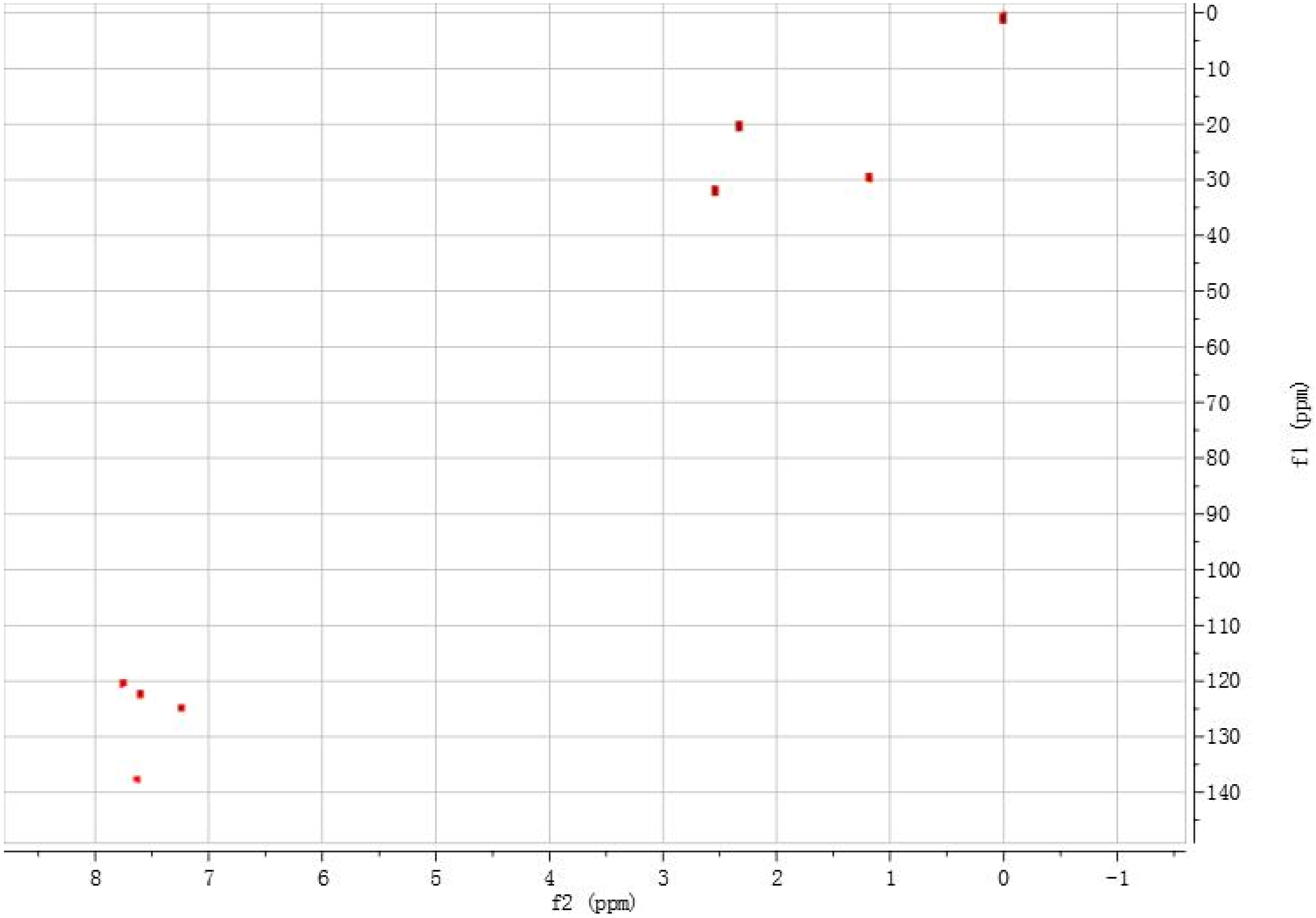

**Figure S14** HMBC spectrum of HLM A (**7**)

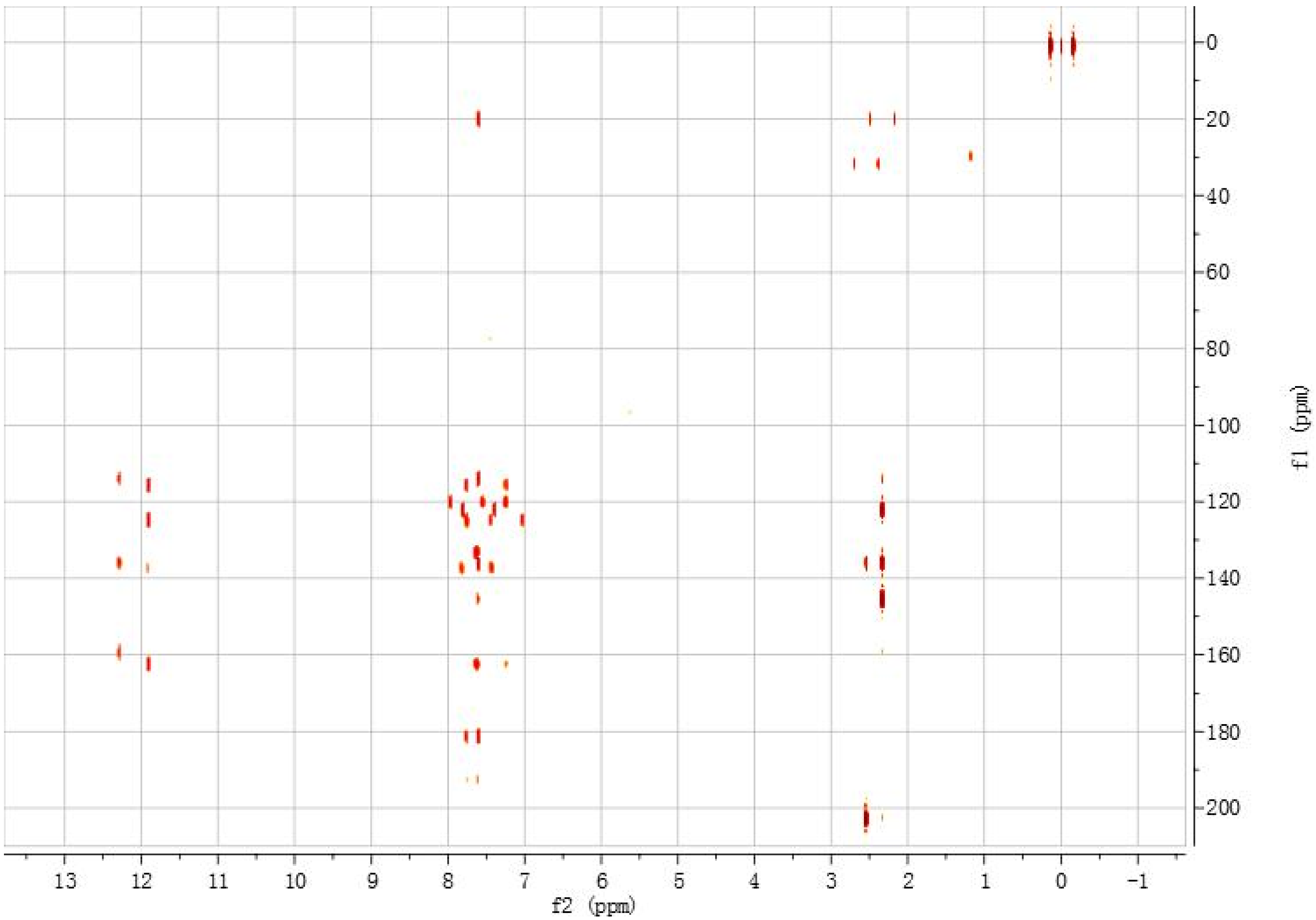

**Figure S15** $^1$H-$^1$H COSY spectrum of HLM A (**7**)

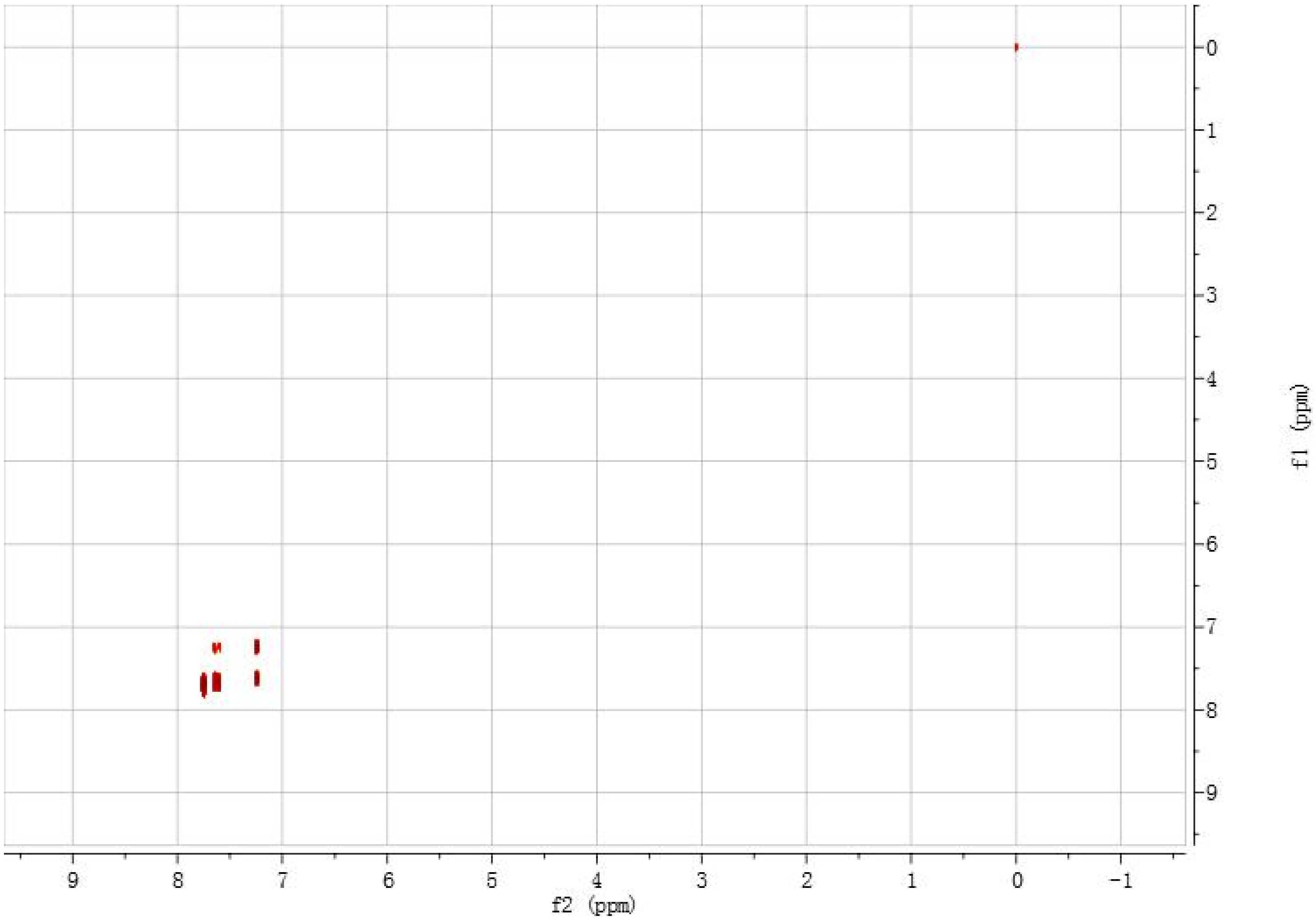

**Figure S16** $^1$H NMR spectrum of HLM B (**8**) in DMSO-$d_6$ (500 MHz)

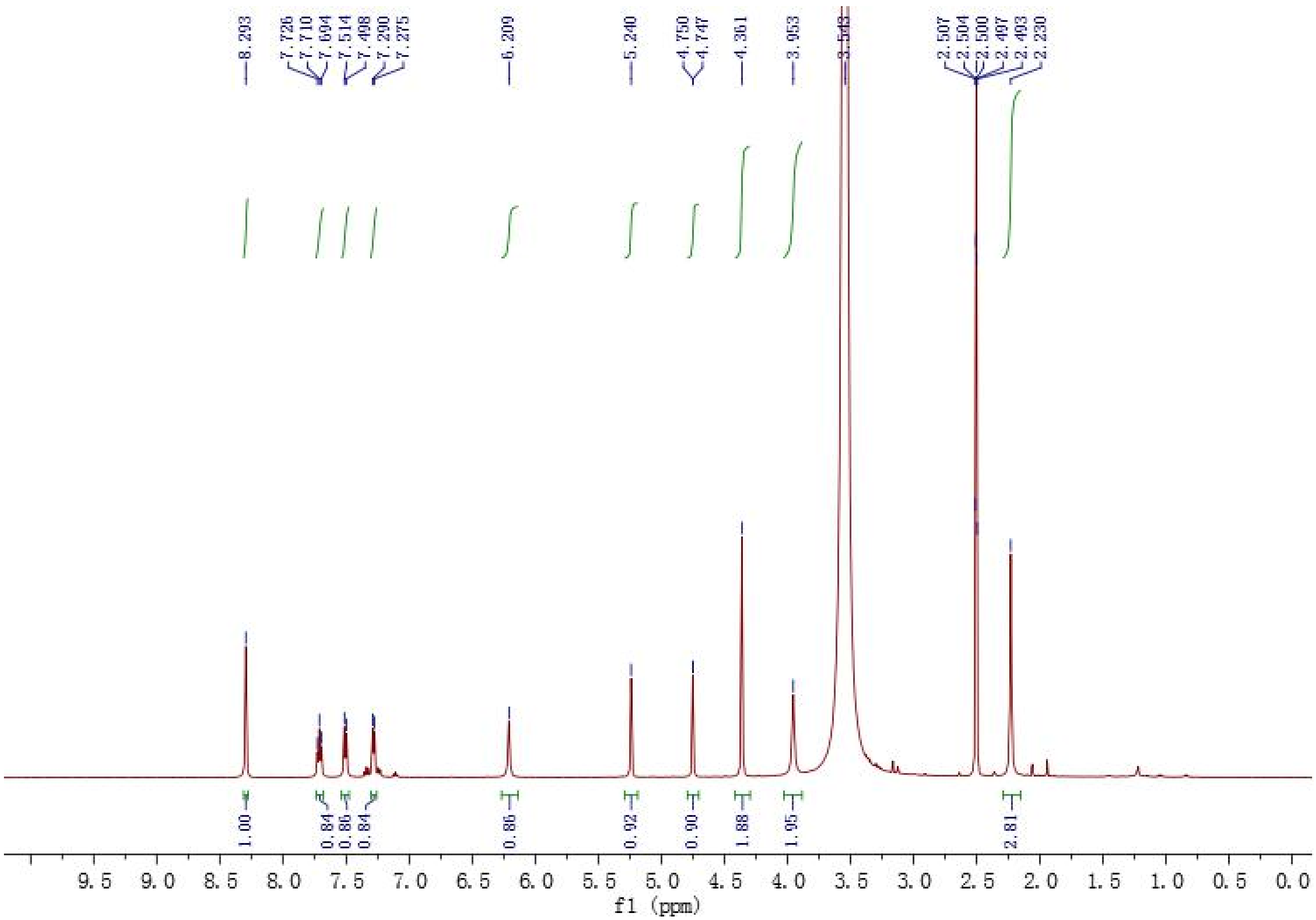

**Figure S17** $^{13}$C NMR spectrum of HLM B (**8**) in DMSO-$d_6$ (125 MHz)

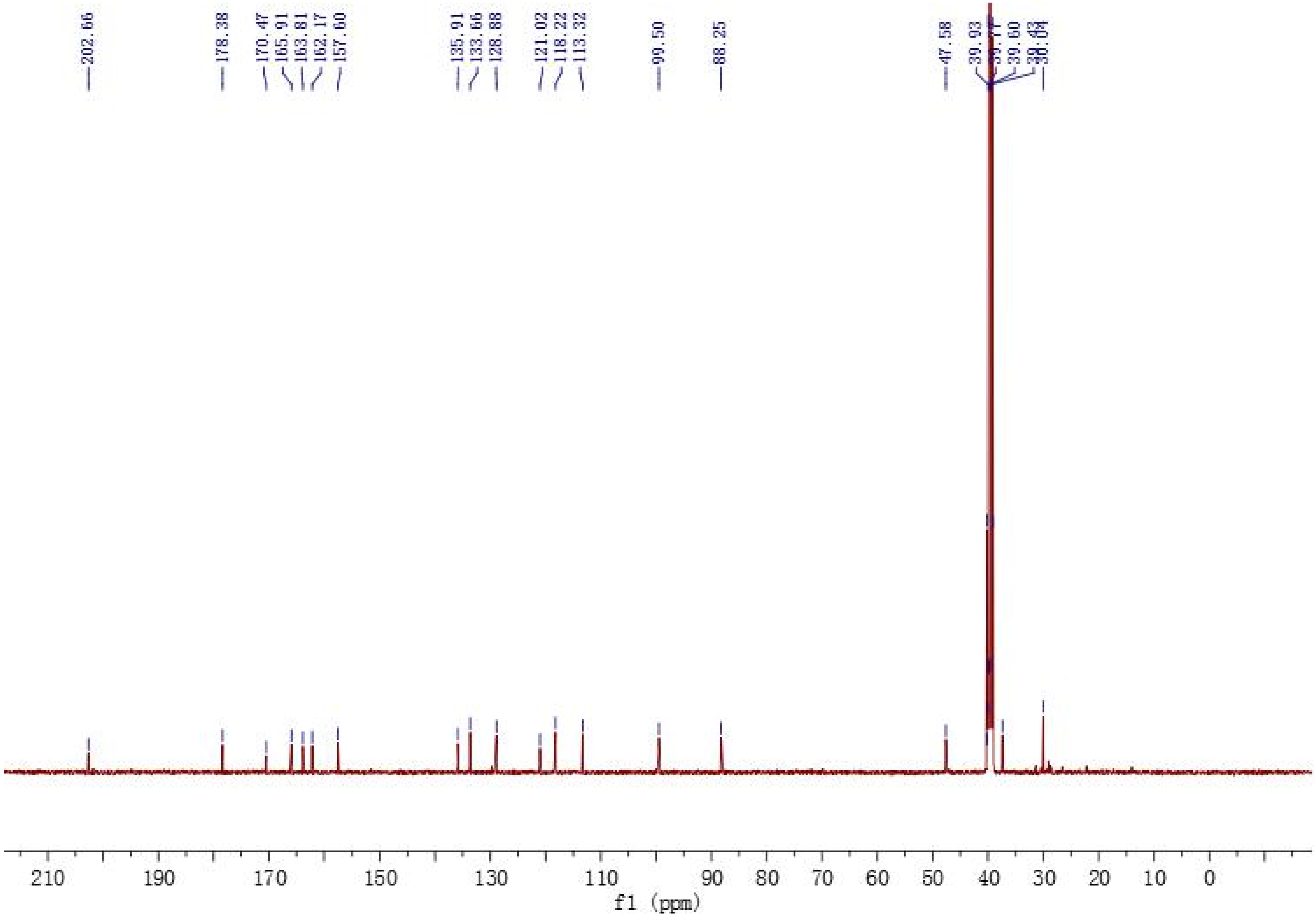

**Figure S18** HSQC spectrum of HLM B (**8**)

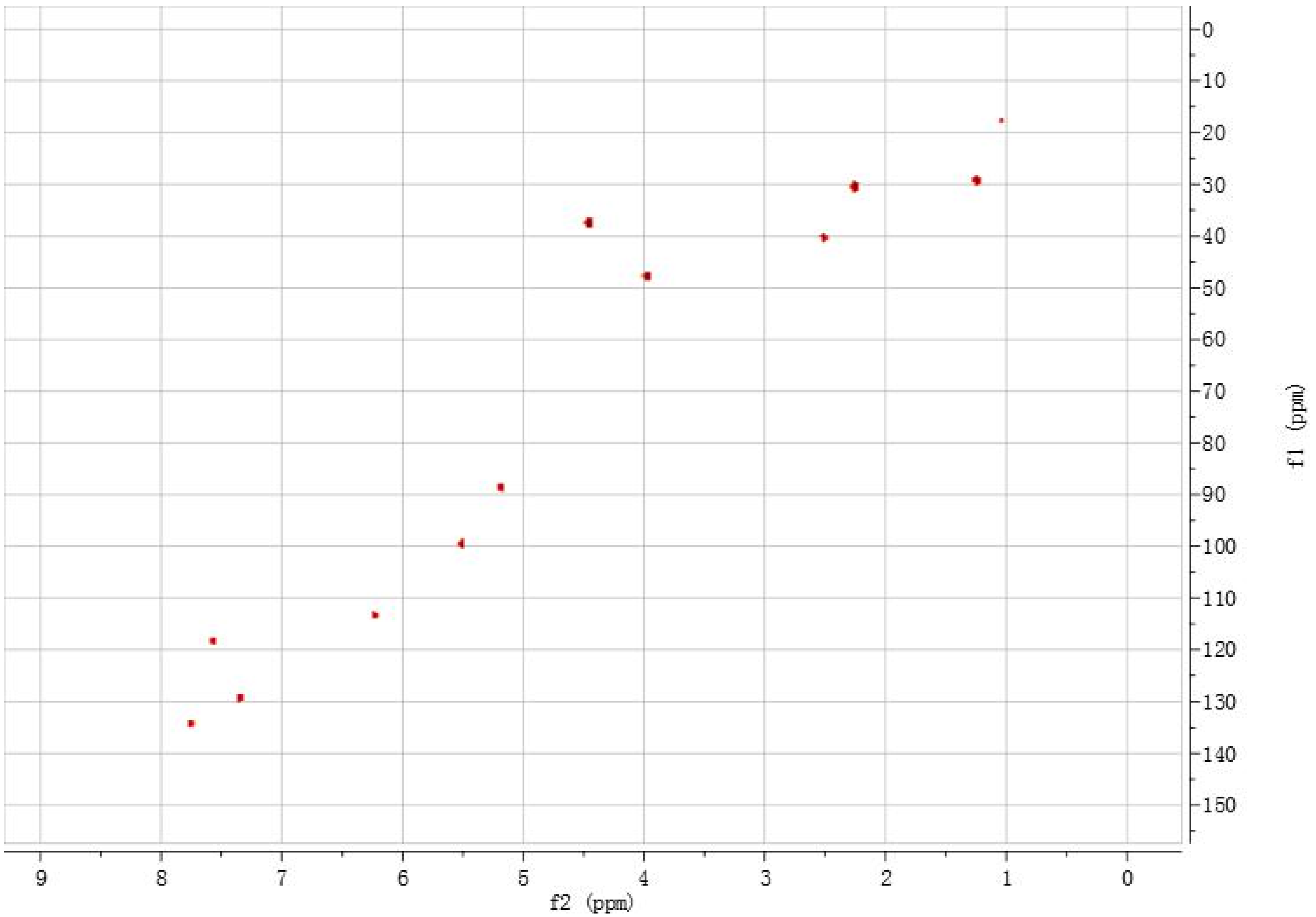

**Figure S19** HMBC spectrum of HLM B (**8**)

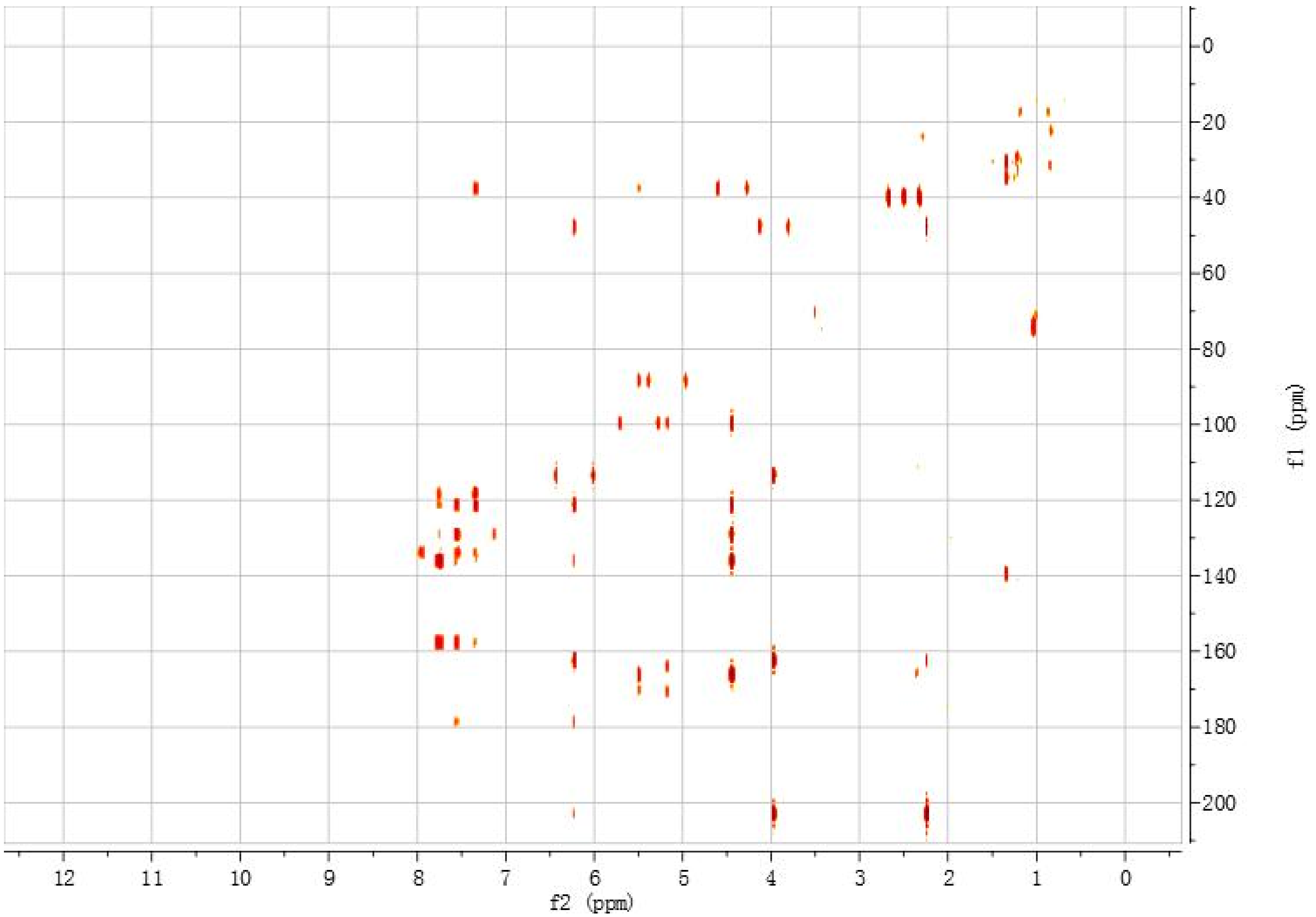

**Figure S20** $^{1}H$-$^{1}H$ COSY spectrum of HLM B (**8**)

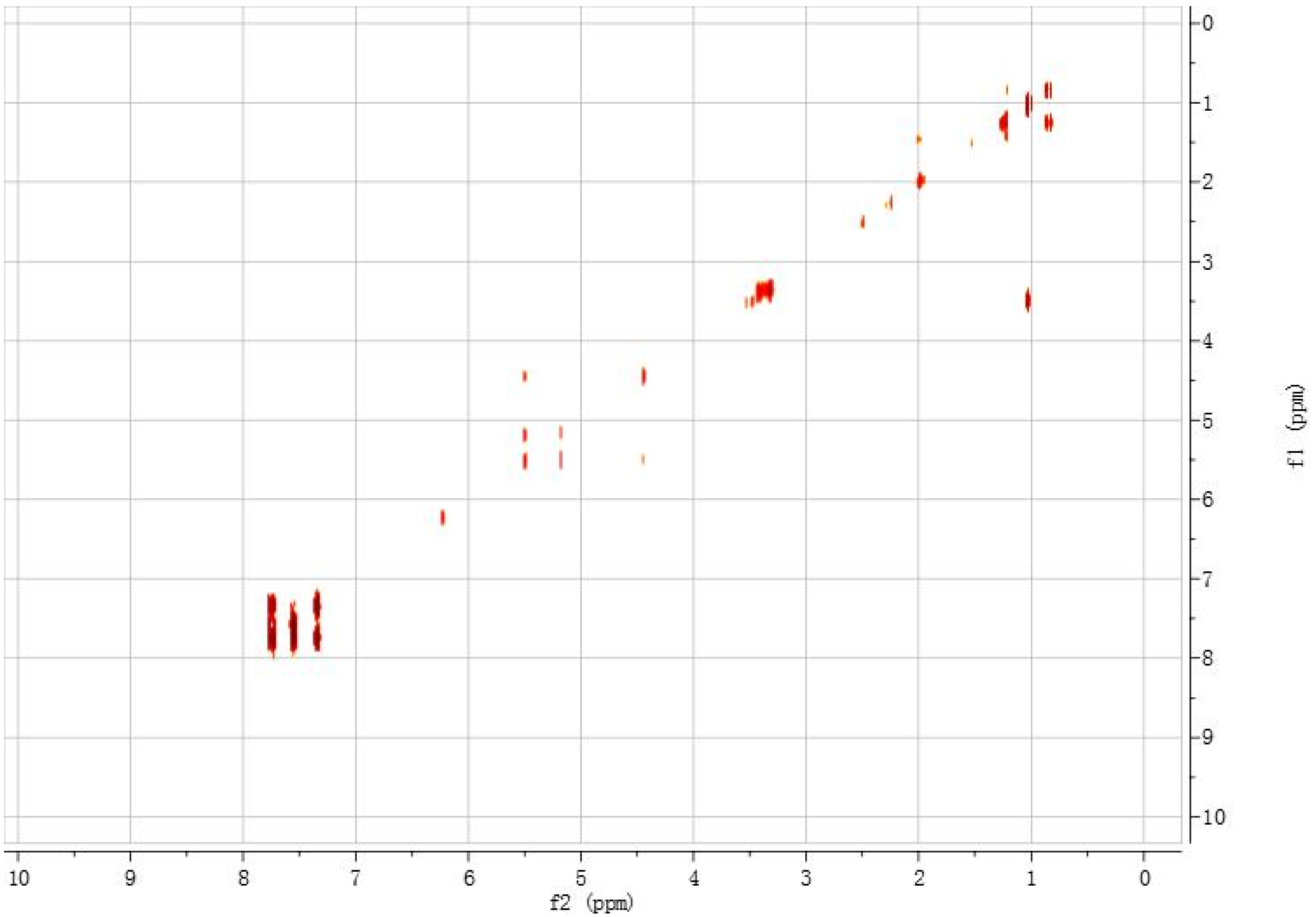

**Figure S21** $^{1}$H NMR spectrum of HLM C (**9**) in DMSO-$d_6$ (500 MHz)

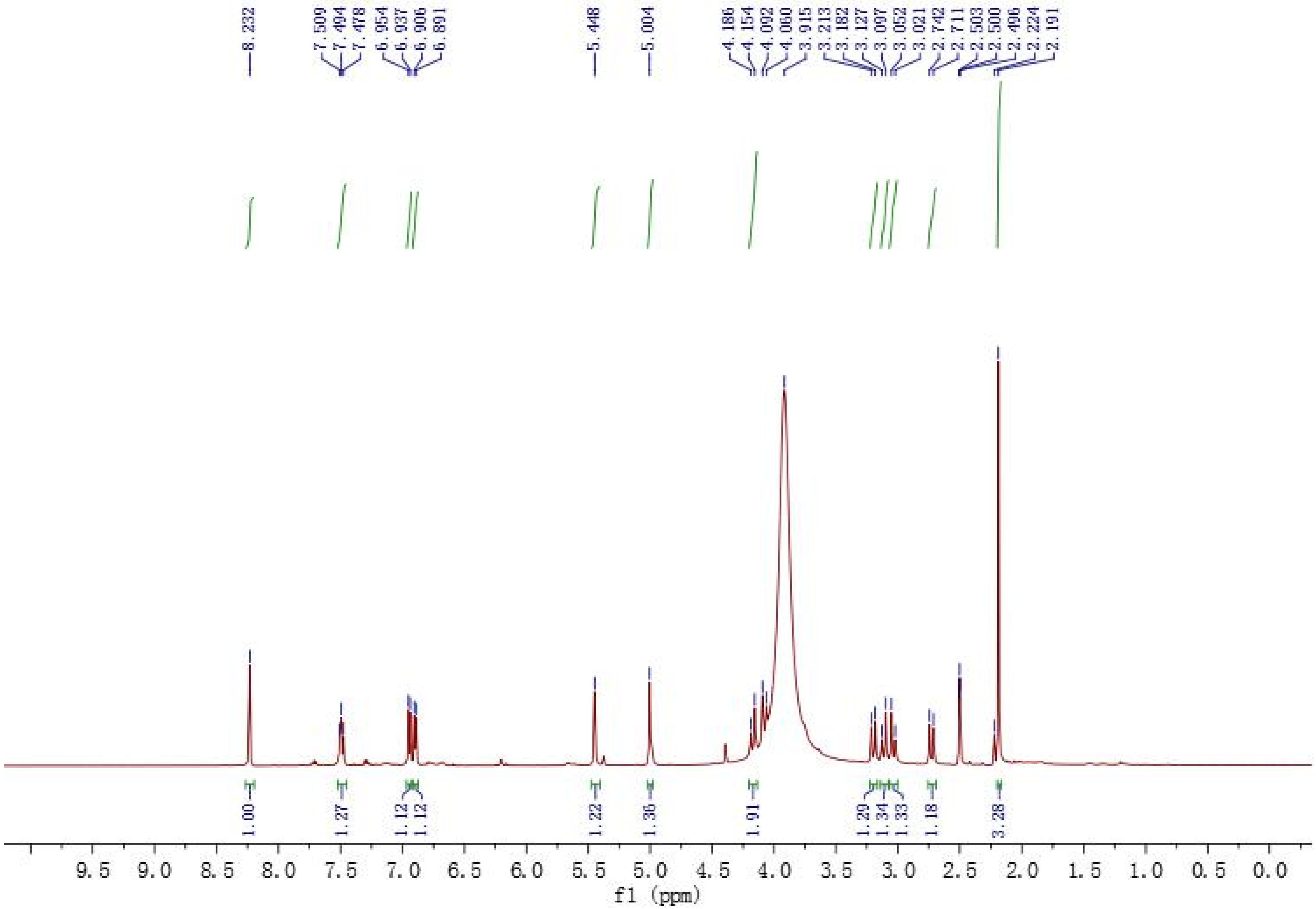

**Figure S22** $^{13}$C NMR spectrum of HLM C (**9**) in DMSO-$d_6$ (125 MHz)

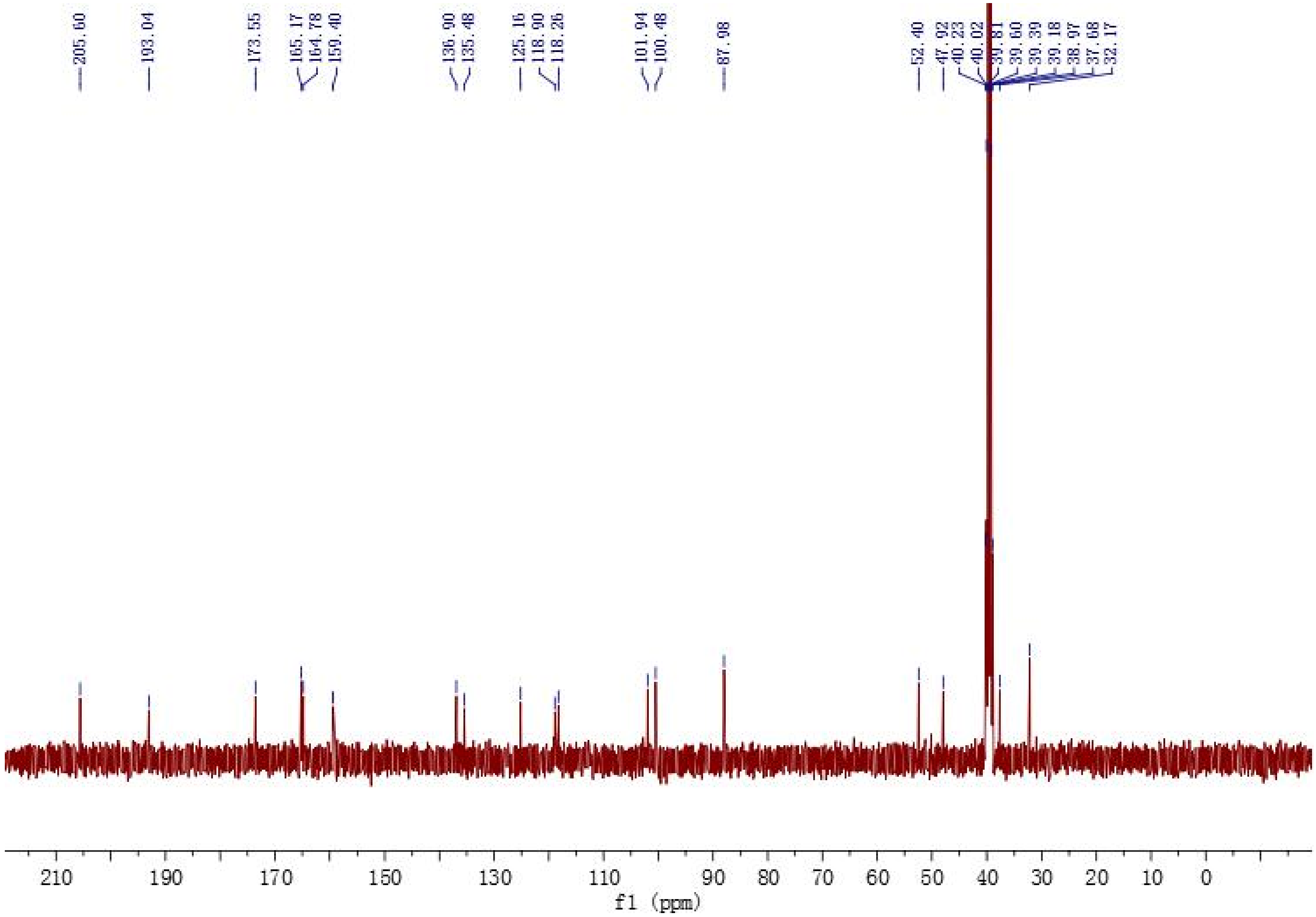

**Figure S23** HSQC spectrum of HLM C (**9**)

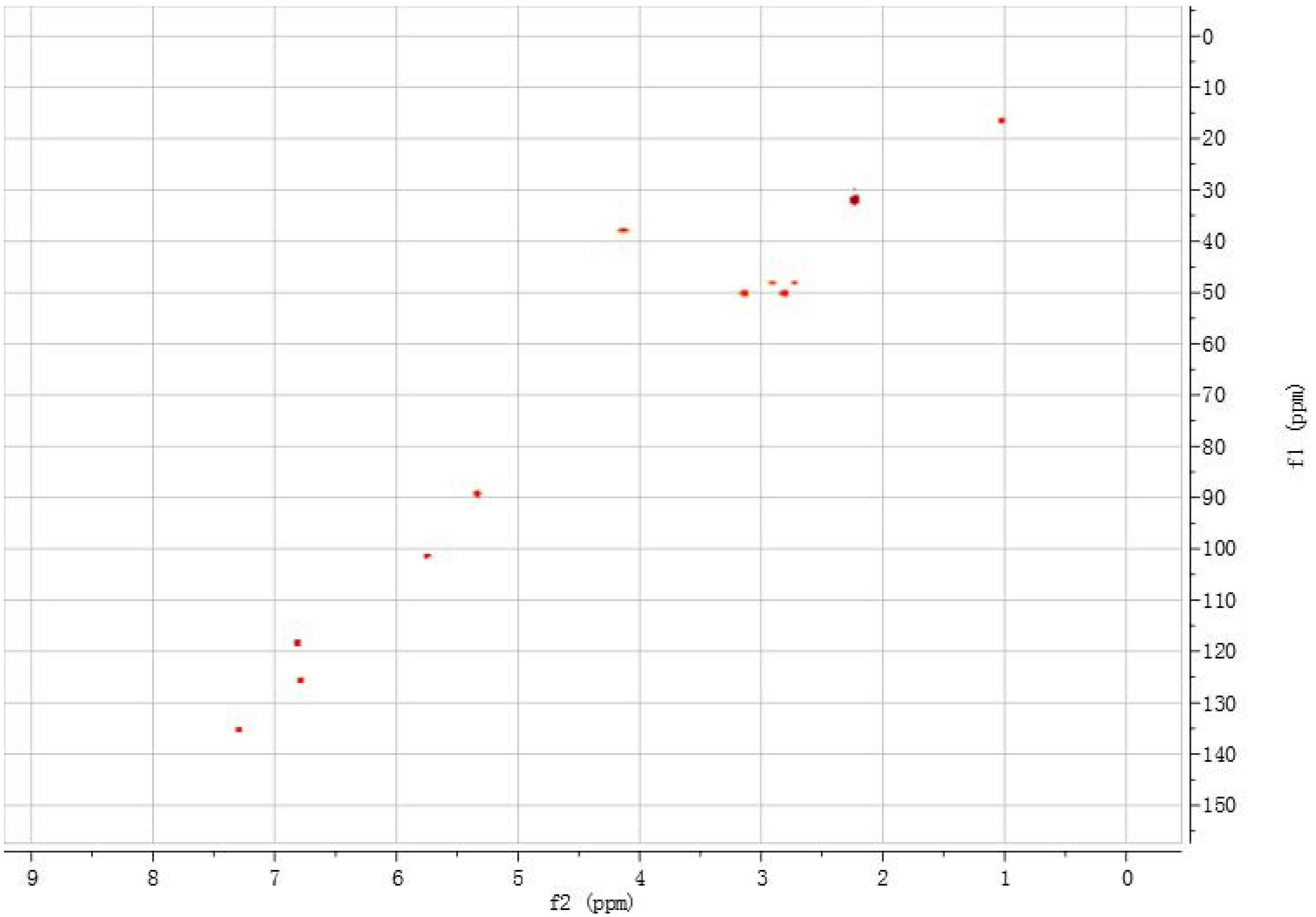

**Figure S24** HMBC spectrum of HLM C (**9**)

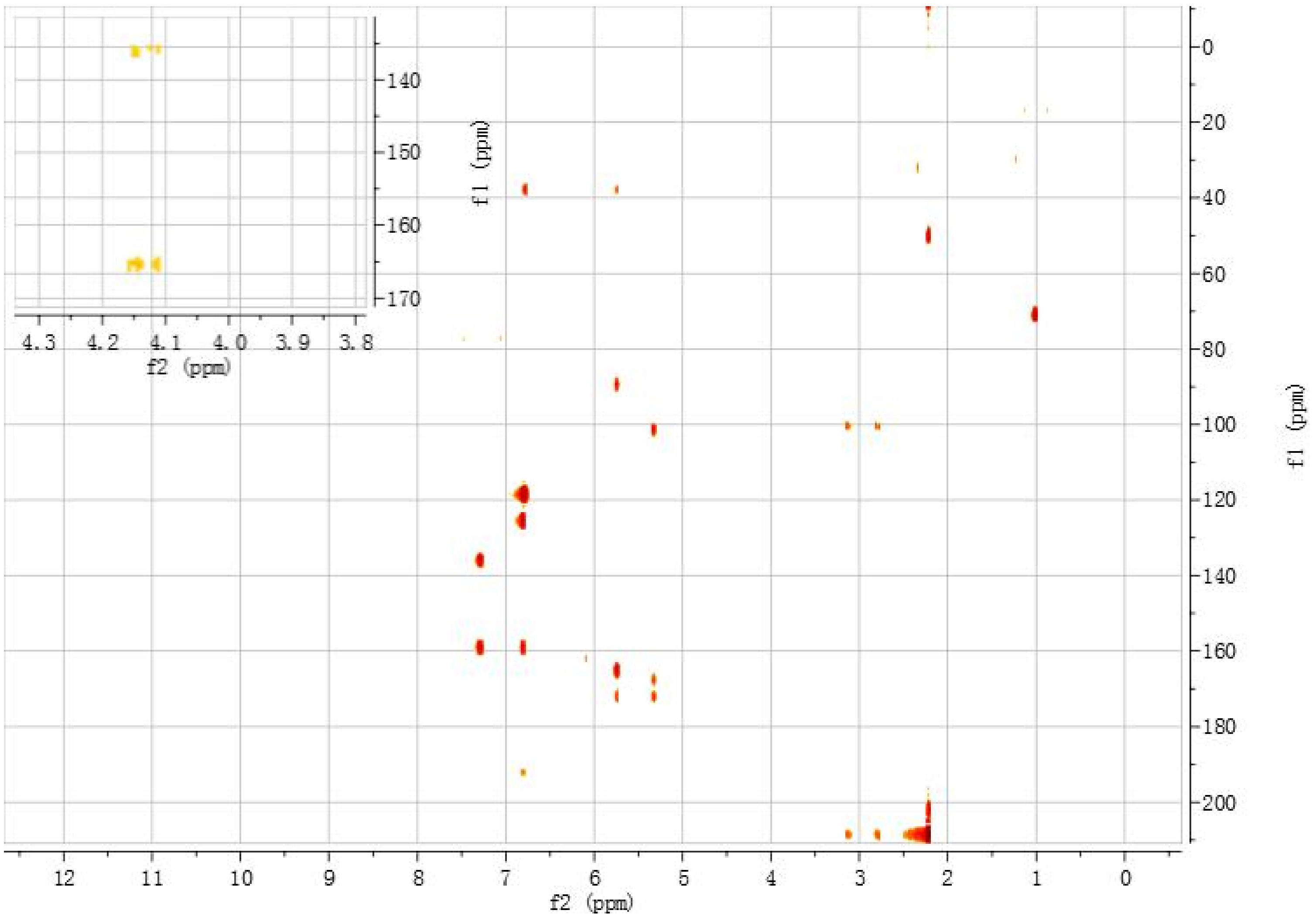

**Figure S25** $^{1}$H-$^{1}$H COSY spectrum of HLM C (**9**)

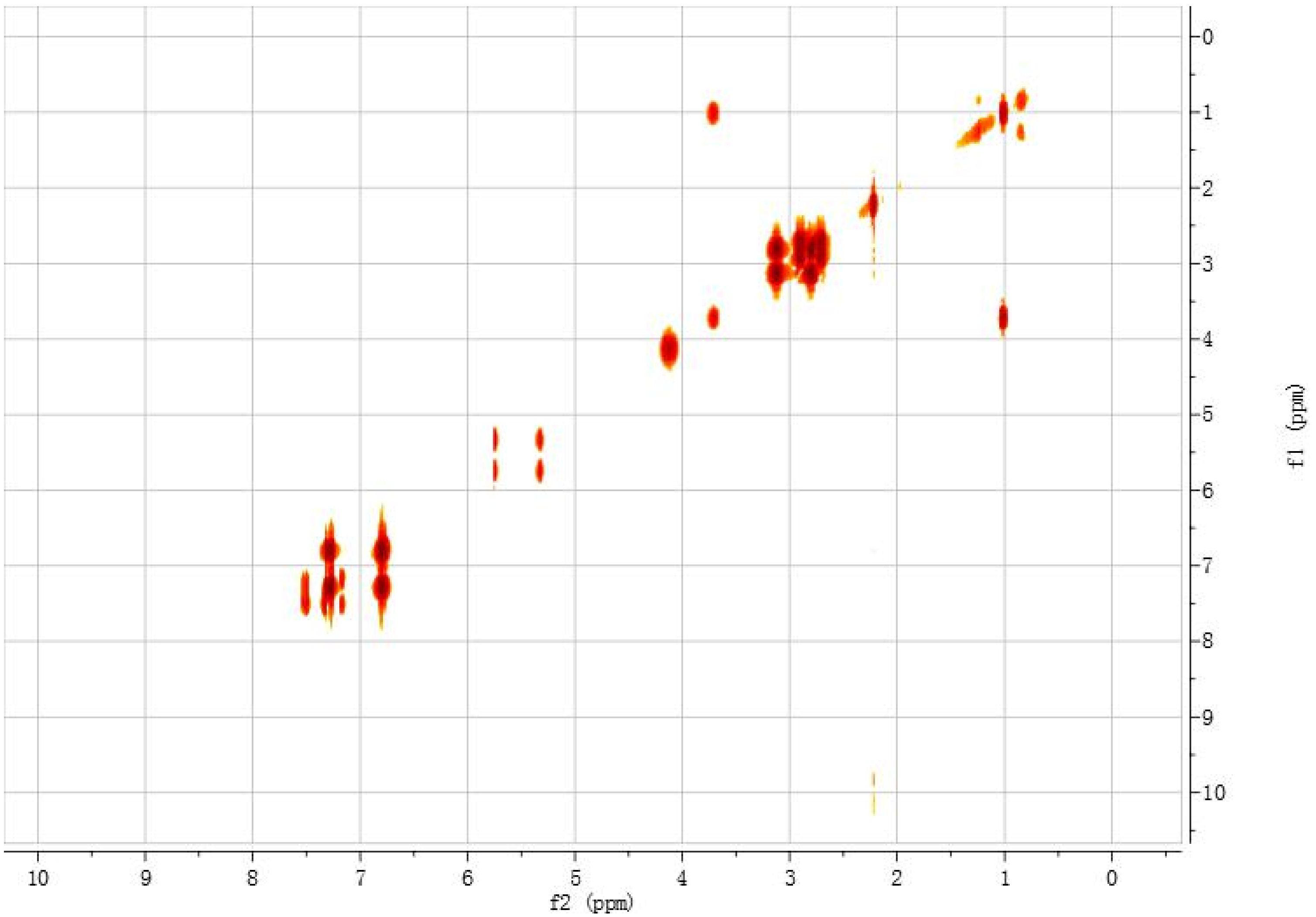

Supplement: Supplementary file 1 [file Presentation_1.PDF]
